# Lure-and-kill macrophage nanoparticles alleviate the severity of experimental acute pancreatitis

**DOI:** 10.1038/s41467-021-24447-4

**Published:** 2021-07-06

**Authors:** Qiangzhe Zhang, Julia Zhou, Jiarong Zhou, Ronnie H. Fang, Weiwei Gao, Liangfang Zhang

**Affiliations:** Department of Nanoengineering, Chemical Engineering Program, Moores Cancer Center, University of California San Diego, La Jolla, CA USA

**Keywords:** Bioinspired materials, Drug delivery

## Abstract

Acute pancreatitis is a disease associated with suffering and high lethality. Although the disease mechanism is unclear, phospholipase A2 (PLA2) produced by pancreatic acinar cells is a known pathogenic trigger. Here, we show macrophage membrane-coated nanoparticles with a built-in ‘lure and kill’ mechanism (denoted ‘MΦ-NP(L&K)’) for the treatment of acute pancreatitis. MΦ-NP(L&K) are made with polymeric cores wrapped with natural macrophage membrane doped with melittin and MJ-33. The membrane incorporated melittin and MJ-33 function as a PLA2 attractant and a PLA2 inhibitor, respectively. These molecules, together with membrane lipids, work synergistically to lure and kill PLA2 enzymes. These nanoparticles can neutralize PLA2 activity in the sera of mice and human patients with acute pancreatitis in a dose-dependent manner and suppress PLA2-induced inflammatory response accordingly. In mouse models of both mild and severe acute pancreatitis, MΦ-NP(L&K) confer effective protection against disease-associated inflammation, tissue damage and lethality. Overall, this biomimetic nanotherapeutic strategy offers an anti-PLA2 treatment option that might be applicable to a wide range of PLA2-mediated inflammatory disorders.

## Abstract (web summary)

Phospholipase A2 (PLA2) inhibitors have been shown to be able to treat acute pancreatitis, but are toxic with systemic application. Here the authors design a nanoparticle with macrophage membrane components to carry PLA2 inhibitor to macrophages and treat pancreatitis in mice, with no evidence of toxicity.

## Introduction

Acute pancreatitis (AP) is a disease featuring the premature activation of digestive enzymes in the pancreatic acinar cells (PACs) that leads to the digestion of the pancreas itself^1,2^. The self-digestive condition provokes inflammation, edema, hemorrhage, and necrosis of the pancreatic tissue. Injured PACs release an array of pro-inflammatory factors, causing peripancreatic tissue destruction and distant organ damage. The primary cause of death is multisystem organ failure resulted from systemic inflammatory response syndrome or the sepsis induced by infections of necrotic tissues^3^. AP is the most common cause of hospitalization for gastrointestinal diseases worldwide, and the overall incidence rate continues to rise^4^. Although most of the cases are mild and self-limiting, about 25% of the patients develop severe AP, and the mortality rate of this patient group reaches 30%^5^. Despite concerted efforts in the past few decades, there is still no effective drug therapy to treat AP^6^. The principles of care remain supportive^7,8^.

Phospholipase A2 (PLA2), specifically group IIA secreted PLA2 isoform, is a known pathogenic trigger of AP^9,10^. The release of PLA2 from the pancreatic tissue damages the cell membrane of surrounding acinar cells, producing pro-inflammatory signals that exacerbate the injury^11,12^. PLA2 secreted from both pancreatic and distant sources propagates a systemic inflammatory response leading to life-threatening complications such as acute respiratory distress syndrome and multisystem organ failure^13,14^. Serum levels of PLA2 correlate with the disease severity of AP patients and have been used to assess the diagnosis and prognosis of the disease^15^. Inhibition of PLA2 was shown to significantly decrease the activity of digestive enzymes and reduce tissue damages in AP^16,17^. Despite the potential, effective PLA2 inhibitors suitable for AP treatment remain unavailable due to the lack of specificity, inherent toxicity, and poor pharmacokinetic profiles^18,19^.

**Fig. 1:**
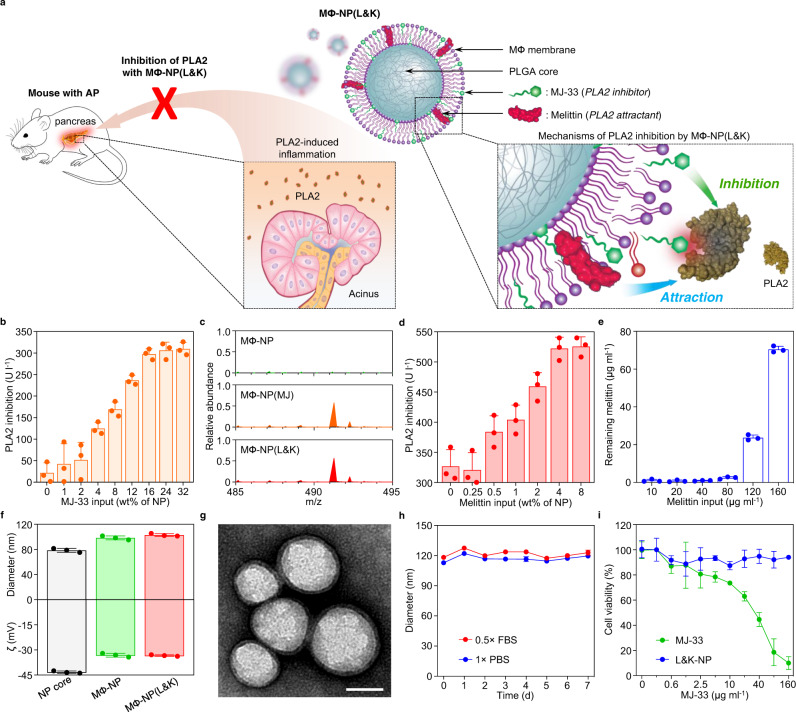
Fabrication and characterization of MΦ-NP(L&K). **a** Schematic representation of MΦ-NP(L&K) designed to inhibit PLA2 during AP progression. **b** Optimization of MJ-33 input into MФ-NP with a PLA2 inhibition assay. **c** Confirmation of MJ-33 loading into MΦ-NP(MJ) using mass spectrometry (MS). **d** Optimization of melittin input into MΦ-NP(MJ) with a PLA2 inhibition assay. **e** Quantitative analysis of melittin loading into MΦ-NP(L&K) with a hemolysis assay. **f** DLS measurements of MΦ-NP(L&K) for hydrodynamic size (diameter, nm) and zeta potential (ζ, mV). **g** A representative image of MΦ-NP(L&K) examined with TEM. Samples were stained with uranyl acetate. Scale bar, 100 nm. **h** Stability of MΦ-NP(L&K) in 1× PBS or 0.5× FBS, assessed by monitoring nanoparticle size (diameter) over 7 d. **i** Comparison of the cytotoxicity of free MJ-33 and MΦ-NP(L&K) loaded with equivalent amounts of MJ-33 on mouse J774A.1 macrophages. In all studies, *n* = 3 independent experiments using the same batch of corresponding nanoparticles. In (**b**, **d**), and (**e**), data presented as mean + s.d.. In (**f**, **h**), and (**i**), data presented as mean ± s.d. Source data are provided as a Source Data file for (**b**–**f**, **h**, **i**).

The unmet needs in AP treatment motivate us to develop nanoparticles with natural cell membrane coating for safe and effective PLA2 inhibition^20^. The membrane coating allows nanoparticles to exploit cellular membrane properties and intercept harmful chemical agents, toxins, enzymes, and viruses for biodetoxification^21–24^. In the pathophysiology of AP, macrophage is an essential target of PLA2 to upregulate pro-inflammatory mediators and altered cytokine reactions^25^. Therefore, we select macrophage (MΦ) membrane as coating materials to attract PLA2 away from the intended host cell target (Fig. 1a). We further dope the membrane with melittin, a short peptide that can lure PLA2 to attack the membrane^23,26^. Meanwhile, to overcome the sacrificial nature of a simple macrophage decoy, we insert MJ-33, a lipid-like PLA2-specific inhibitor into the membrane. The membrane embedment suppresses the toxicity of the inhibitor molecules otherwise unsafe for AP treatment. Upon PLA2 attack, the inhibitors are exposed and will bind and kill PLA2. With such a design, the nanoparticles (denoted ‘MΦ-NP(L&K)’) leverage macrophage membrane functions and orchestrate a ‘lure and kill’ mechanism for safe and responsive inhibition of PLA2.

In this work, we report MΦ-NP(L&K) formulation according to the above design principles and test therapeutic efficacy in mouse models of AP. We show that the nanoparticle formulation eliminates the toxicity associated with free melittin and MJ-33. In vitro, we demonstrate that MΦ-NP(L&K) suppresses PLA2 activity in sera of mice and humans of AP. In vivo, by using mouse models of both mild and severe AP, we show that MΦ-NP(L&K) can effectively protect the pancreas and reduce disease severity. Overall, MΦ-NP(L&K) are a unique biomimetic nanoparticle platform for effective and safe PLA2 inhibition with the potential for treating AP and other PLA2-mediated diseases.

## Results and discussion

### Nanoparticle preparation and characterization

To synthesize MΦ-NP(L&K), the cell membrane was first derived from macrophages^27^. Then MJ-33 was incorporated into the cell membrane through sonication (Supplementary Fig. 1). The membrane was subsequently coated onto poly(lactic-*co*-glycolic acid) (PLGA) cores to form MJ-incorporated macrophage membrane-coated nanoparticles (denoted ‘MФ-NP(MJ)’). Then, the suspension of MФ-NP(MJ) was added with melittin, which spontaneously inserted into the membrane of the nanoparticles, leading to the final MΦ-NP(L&K) formulation^22^. During the formulation, increasing MJ-33 input caused a higher inhibition of PLA2, but the effect plateaued at an input of 16 wt% (Fig. 1b). The incorporation of MJ-33 into MФ-NP(MJ) and MΦ-NP(L&K) was confirmed by using mass spectrometry (MS). Characteristic molecular ion peak was identified at m/z = 491.3, which was in agreement with the theoretical molecular weight of MJ-33, indicating successful loading of MJ-33 into the nanoparticle formulations (Fig. 1c). Using a calibration curve constructed from MJ-33 standards, an MJ-33 input of 16 wt% was calculated to achieve a loading yield of 9.4 wt%, which was used for all subsequent MФ-NP(MJ) formulation (Supplementary Fig. 2). Similarly, increasing melittin input also led to an increase of PLA2 inhibition (Fig. 1d). In this case, a plateau appeared at a 4 wt% input, equivalent to a 3.9 wt% melittin loading yield, calculated by quantifying the remaining melittin after incubation with MФ-NP(MJ) (Fig. 1e and Supplementary Fig. 3). This input was used in all subsequent MΦ-NP(L&K) formulations. When examined with dynamic light scattering (DLS), MΦ-NP(L&K) showed a hydrodynamic diameter larger than that of the uncoated PLGA cores with a less negative surface zeta potential, consistent with the addition of a membrane bilayer structure (Fig. 1f). Notably, values of the diameter and zeta potential of MΦ-NP(L&K) were comparable to those of nanoparticles coated with macrophage membrane alone (denoted ‘MФ-NP’), indicating negligible impacts from membrane incorporation of MJ-33 and melittin. When visualized with transmission electron microscopy (TEM), MΦ-NP(L&K) showed a core–shell structure that indicated a unilamellar membrane coating around the polymeric core (Fig. 1g)^22,28^. The membrane coating bestowed MΦ-NP(L&K) with extended colloidal stability in both 1× phosphate-buffered saline (PBS) and 0.5× fetal bovine serum (FBS) (Fig. 1h). Whereas free MJ-33 was shown to be toxic to macrophages, MΦ-NP(L&K) with equivalent MJ-33 were nontoxic (Fig. 1i), suggesting a strong association of MJ-33 to the nanoparticles that prevented its participation in cell signaling for toxicity. A series of quality assurance specifications were established to ensure the physicochemical and biological reproducibility of fabricating MΦ-NP(L&K) (Supplementary Table 1).

**Fig. 2:**
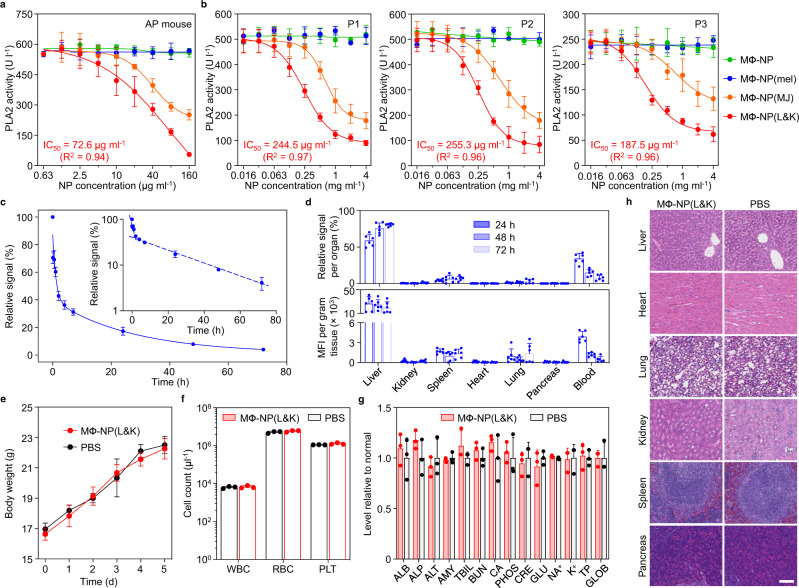
PLA2 inhibition capacity and in vivo characterizations. Comparison of the inhibition capacity of MФ-NP, MΦ-NP(mel), MΦ-NP(MJ), and MΦ-NP(L&K) against PLA2 in serum from mice with acute pancreatitis (**a**) and human patients with acute pancreatitis (**b**). **c** Pharmacokinetics of MΦ-NP(L&K). DiR-loaded nanoparticles were injected intravenously through the tail vein of mice. At various timepoints, blood was withdrawn intraorbitally and measured for fluorescence at 780 nm to evaluate the systemic circulation lifetime of the nanoparticles (inset, semi-log plot of fluorescence at various timepoints). **d** Biodistribution of MΦ-NP(L&K). Fluorescently labeled nanoparticles were injected intravenously into the mice. At each timepoint (24, 48, and 72 h), the organs from a randomly grouped subset of mice were collected, homogenized, and quantified for fluorescence (top: relative signal per organ, bottom: fluorescence intensity per gram of tissue). **e**–**h** Safety of MΦ-NP(L&K). Healthy mice received intravenous injections of PBS or MΦ-NP(L&K) (80 mg/kg) daily on days 1–4. **e** Body weights of mice taken from day 0 to day 5. **f** Blood cell counts measured on day 5. WBC white blood cells, RBC red blood cells, PLT platelets. **g** Comprehensive blood chemistry panel performed on day 5. ALB albumin, ALP alkaline phosphatase, ALT alanine transaminase, AMY amylase, TBIL total bilirubin, BUN blood urea nitrogen, CA calcium, PHOS phosphorus, CRE creatinine, GLU glucose, NA+ sodium, K+ potassium, TP total protein, GLOB globulin (calculated). **h** H&E-stained histological sections from major organs on day 5. Scale bar, 100 μm. Data presented as mean ± s.d. In all in vitro studies, *n* = 3 independent experiments using the same batch of MΦ-NP(L&K). IC_50_ values were derived from the variable slope model with Graphpad Prism 8. In circulation lifetime and biodistribution studies, *n* = 6 using the same batch of MΦ-NP(L&K). In systemic toxicity studies, *n* = 3 independent experiments using the same batch of MΦ-NP(L&K). In (**f**, **g**), the statistical analysis was performed by using the two-tailed paired Student’s *t*-test with Microsoft Excel. The difference between PBS and MΦ-NP(L&K) groups was not statistically significant (*P* = 0.38, 0.37, and 0.21 for comparison of counts of WBC, RBC, PLT, respectively). The difference of blood chemistry markers was not significant (Supplementary Table 2). Source data are provided as a Source Data file for (**a**–**g**).

Next, MΦ-NP(L&K) were examined for their anti-PLA2 activity in vitro. They were added to serum samples collected from mice with caerulein-induced acute pancreatitis (CAE-AP) that contained elevated levels of PLA2 (Fig. 2a). MΦ-NP(L&K) showed a clear inhibitory effect against PLA2, with an IC_50_ value (half-maximal inhibitory concentration) of 72.6 μg/ml. The inhibition capacity measured from MФ-NP(MJ) was lower (IC_50_ = 110.2 μg/ml), attributable to the absence of melittin, which helps to potentiate the inhibition. Meanwhile, MФ-NP and MФ-NP(mel) (melittin-incorporated MФ-NP) were unable to inhibit PLA2 in the serum samples. The inhibitory effect of MΦ-NP(L&K) was also tested on human serum samples from patients with AP. Similarly, MΦ-NP(L&K) effectively inhibited PLA2 activity in samples from all three patients (IC_50_ = 245, 255, and 188 μg/ml, Fig. 2b). In contrast, a weaker inhibition was observed with MФ-NP(MJ) (IC_50_ = 630, 673, and 614 μg/ml, respectively), and no inhibition was observed with MФ-NP and MФ-NP(mel). Overall, these results demonstrated a ‘lure and kill’ mechanism through the interplay among lipid membrane, melittin, and MJ-33 that together suppressed PLA2 activity. Toward systemic applications, the circulation time of MΦ-NP(L&K) was measured with fluorescently labeled nanoparticles after intravenous administration (Fig. 2c). At 24 and 48 h, MΦ-NP(L&K) showed 17.4% and 8.0% retention in the blood, respectively. Based on a two-compartment model, the elimination half-life was calculated as 18.9 h, comparable to the value measured from MФ-NP^27^. The in vivo tissue distribution of MΦ-NP(L&K) was also evaluated (Fig. 2d). When analyzed per organ, MΦ-NP(L&K) distributed mostly in the blood and the liver. When normalized per gram of tissue, the nanoparticles were largely contained in the liver and spleen, two primary organs of the reticuloendothelial system. Meanwhile, significant fluorescence was also observed in the blood. As the blood fluorescence decreased, a corresponding increase in signal was observed in the liver, indicating the uptake of MΦ-NP(L&K) by the reticuloendothelial system over time^29^. To evaluate the safety of MΦ-NP(L&K), PBS or MΦ-NP(L&K) (80 mg kg^−1^) was intravenously injected into healthy mice daily for 4 days. During the course of the study, the change of body weight in mice receiving MΦ-NP(L&K) was comparable to that of the mice receiving PBS (Fig. 2e). Blood cell count and a comprehensive metabolic panel were performed 24 h after the last nanoparticle injection (Fig. 2f, g, Supplementary Table 2). Compared to mice receiving PBS only, mice receiving MΦ-NP(L&K) showed the absence of toxicity in major organs, including the liver, heart, lungs, kidney, spleen, and pancreas (Fig. 2h). Together, these results indicate a high safety profile for the MΦ-NP(L&K).

### Inhibition of pancreatitis-associated inflammation and acinar cell injury

**Fig. 3:**
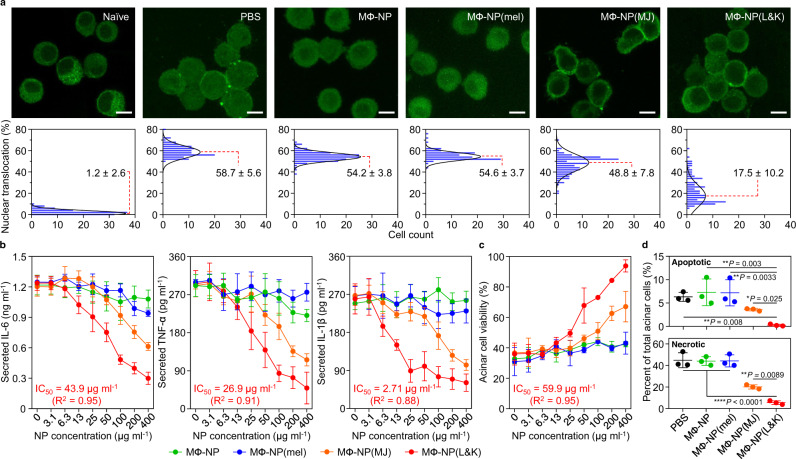
Suppression of PLA2-induced inflammatory response in vitro. **a** Effects of MΦ-NP(L&K) and control formulations on NF-κB nuclear translocation of PLA2-stimulated macrophages. Representative fluorescence images (top row, scale bar, 10 μm) and quantification of nuclear translocation (bottom row) based on corresponding fluorescence images. **b** Effects of MΦ-NP(L&K) and control formulations on IL-6, TNF-α, and IL-1β production from PLA2-stimulated macrophages. **c** Effects of MΦ-NP(L&K) and control formulations on cell viability of PLA2-stimulated pancreatic acinar cells. **d** Effects of MΦ-NP(L&K) and control formulations on apoptosis and necrosis analyzed by flow cytometry using annexin V/PI double staining. Results were analyzed by using one-way ANOVA with Dunnett’s post hoc analysis in Graphpad Prism. Data presented as mean ± s.d. In all datasets, *n* = 4 independent experiments using the same batch of MΦ-NP(L&K). IC_50_ and EC_50_ values were derived from the variable slope model using Graphpad Prism 8. Source data are provided as a Source Data file for (**a**–**d**).

MΦ-NP(L&K) were examined for their ability to inhibit pancreatitis-associated NF-κB activation in macrophages (Fig. 3a)^30^. When naive macrophages were stained for p65, a subunit of NF-κB, higher fluorescence intensity was observed in the cytoplasm relative to that in the nuclear zone, corresponding to a low nuclear translocation (1.2%). In contrast, in macrophages exposed to the serum of CAE-AP mice, p65 appeared in both the cytosol and the nuclear zone. In this case, the nuclear translocation increased significantly (58.7%), indicating NF-κB activation. Macrophages exposed to the serum mixed with MΦ-NP(L&K) showed a reduced p65 distribution in the nuclear zone in relation to that in the cytosol. The lower nuclear translocation (17.5%) implied inhibition of NF-κB activation by the MΦ-NP(L&K). In comparison, macrophages exposed to the serum mixed with control nanoparticles, including MФ-NP, MФ-NP(mel), or MФ-NP(MJ), all exhibited pronounced NF-κB activation (54.2, 54.6, and 48.8% of the nuclear translocation, respectively). Next, MΦ-NP(L&K) were examined for their ability to inhibit of pro-inflammatory cytokine production in macrophages (Fig. 3b)^31^. Macrophages exposed to the serum of CAE-AP mice produced a higher level of IL-6, but the production was inhibited when the serum was also added with MΦ-NP(L&K). The inhibition increased as the concentration of MΦ-NP(L&K) increased. At all concentrations tested, MΦ-NP(L&K) inhibited IL-6 production more than all control formulations. In addition to IL-6, analysis on additional pro-inflammatory cytokines, including TNF-α and IL-1β, also showed similar inhibition effects.

Acinar cell death is a hallmark of AP^32^, and MΦ-NP(L&K) were thus examined for their ability to protect PACs. Exposure to the serum of CAE-AP mice significantly reduced the viability of PACs (Fig. 3c). However, viability increased when MΦ-NP(L&K) were added to the PACs, indicating a protective effect against cell death. The cell viability increased as the nanoparticle concentration increased, corresponding to an EC_50_ (half-maximal effective concentration) value of 59.9 μg/ml. A dose-dependent increase of PAC viability was also observed when MФ-NP(MJ) were added, but a lower EC_50_ value was obtained (363 μg/ml). PACs added with MФ-NP and MФ-NP(mel) showed viability no higher than 50%, suggesting that they were ineffective in protecting PACs from the serum. The protection was further analyzed for APC necrosis and apoptosis (Fig. 3d and Supplementary Fig. 4). Exposure to the serum of CAE-AP mice resulted in significant portions of necrotic and apoptotic cells. When MΦ-NP(L&K) were added to the cells, the percentages of both cell populations were reduced, confirming the nanoparticles were able to protect against cell death. Similarly, MФ-NP(MJ) showed partial protection of the APCs, whereas MФ-NP and MФ-NP(mel) showed negligible protection.

### Protection of mice with mild acute pancreatitis

**Fig. 4:**
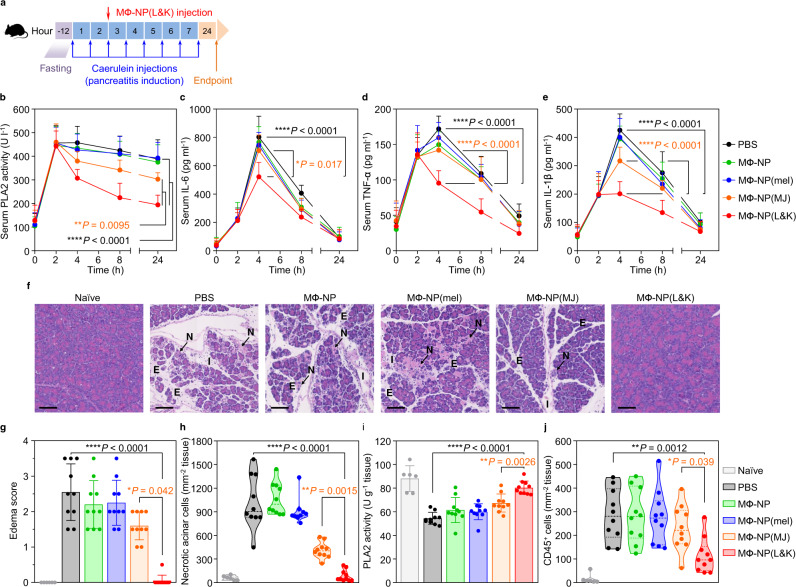
Therapeutic efficacy in a mouse model of mild acute pancreatitis. **a** The study protocol of pancreatitis induction and treatment with MΦ-NP(L&K). **b** PLA2 activity profile in serum of CAE-AP mice treated with different nanoparticle formulations. Concentration profiles of key inflammatory cytokines, including IL-6 (**c**), TNF-α (**d**), and IL-1β (**e**), in the serum of CAE-AP mice treated with different nanoparticle formulations. **f** Representative images of H&E staining on pancreas sections from CAE-AP mice collected 24 h after receiving the treatments. Scale bars, 100 μm. N acinar necrosis, E edema, I cell infiltration. **g** Edema score of H&E-stained pancreas sections from CAE-AP mice after receiving different nanoparticle treatments. **h** Quantification of necrotic cells in the H&E-stained pancreas sections from CAE-AP mice receiving different nanoparticle treatments. **i** PLA2 activity in the pancreatic tissue of CAE-AP mice treated with different nanoparticle formulations. **j** Quantification of CD45^+^ cells in the anti-CD45-stained pancreas sections from CAE-AP mice receiving different nanoparticle treatments. For serum PLA2 and cytokine profiles, statistical analysis was performed using repeated-measure one-way ANOVA with Dunnett’s post hoc analysis. Statistical difference in edema scores was analyzed by Kruskal–Wallis non-parametric test with Dunnett’s post hoc analysis. Necrotic cell counts, tissue PLA2 activity, and CD45^+^ cell counts were analyzed by using one-way ANOVA with Dunnett’s post hoc analysis. Data presented as mean ± s.d. *P ≤ 0.05, **P ≤ 0.01, ***P ≤ 0.001, ****P ≤ 0.0001. In all datasets, *n* = 10 mice treated with the same batch of MΦ-NP(L&K). Source data are provided as a Source Data file for (**b**–**e**, **g**–**j**).

MΦ-NP(L&K) were further evaluated for their protective effects in a mouse model of mild acute pancreatitis (CAE-AP) induced by intraperitoneal injections of caerulein^33^. Following AP induction, MΦ-NP(L&K) or control formulations, including MФ-NP, MФ-NP(mel), MФ-NP(MJ), and PBS, were injected intravenously through the mouse tail vein for efficacy evaluation (Fig. 4a). First, the treatment efficacy was evaluated at a systemic level by examining serum levels of PLA2 and pro-inflammatory cytokines, including IL-6, TNF-α, and IL-1β, for 24 h during the disease progression (Fig. 4b–e). In the study, the injection of caerulein led to a rapid increase of serum PLA2 level, which reached a maximum in 2 h and then gradually decreased. Throughout the study, the PLA2 level in mice injected with MΦ-NP(L&K) (40 mg/kg, Supplementary Fig. 5) remained significantly lower compared to PLA2 levels in all other groups treated with control formulations, indicating an inhibitory effect against AP development. Meanwhile, the injection of caerulein also boosted serum cytokine levels, which reached maximum in 4 h and then started to decrease. The levels of these cytokines in mice injected with MΦ-NP(L&K) remained significantly lower compared to those in mice injected with control formulations, further confirming the inhibition of AP by MΦ-NP(L&K). Notably, MΦ-NP(L&K) showed a significantly higher inhibition than MФ-NP(MJ), suggesting the role of melittin in further potentiating anti-PLA2 effects.

Next, the efficacy of MΦ-NP(L&K) was evaluated at a tissue level by examining the pathological changes of the pancreas during the treatment. In the study, mice were sacrificed 24 h after the initial AP induction. The pancreas was harvested for histopathological analysis. As shown in Fig. 4f, H&E-stained pancreatic sections from healthy naive mice showed tight interlobular and intralobular spaces, with the absence of acinar necrosis or immune infiltration. However, tissue sections from CAE-AP mice injected with PBS exhibited diffuse widening of interlobular and intralobular spaces (edema), marked acinar necrosis, and evident cell infiltration into the interlobular spaces. Similar features were also observable in sections from CAE-AP mice injected with MФ-NP, MФ-NP(mel), or MФ-NP(MJ), indicating a lack of efficacy from these formulations. In contrast, the tissue of CAE-AP mice injected with MΦ-NP(L&K) appeared similar to that of healthy naive mice without obvious acinar necrosis or immune infiltration, demonstrating clear protection of the pancreas during AP. Comprehensive histopathological analysis was performed on sections from all animals in the study (Supplementary Figs. 6–8)^34^. Based on such analysis, the degree of pancreatic edema was evaluated by scoring the extent of interlobular and intralobular space widening in the pancreatic sections (Fig. 4g and Supplementary Figs. 7–12). While mice injected with PBS received an edema score of 2.6 ± 0.8, those injected with MΦ-NP(L&K) had a significantly reduced score of 0.1 ± 0.1, comparable to that of the healthy naive mice. Scores from mice injected with control nanoparticles remained significantly higher, indicating a lack of protection on the pancreas. Furthermore, counts of necrotic acinar cells were elevated in CAE-AP mice injected with PBS, but remained at the basal levels in those injected with MΦ-NP(L&K) (Fig. 4h). Notably, mice injected with MФ-NP(MJ) showed a partial reduction of necrotic acinar cell count, likely due to the presence of the inhibitor without melittin. The pancreatic tissues were also examined for PLA2 activity (Fig. 4i). At 24 h after the initial AP induction, mice injected with PBS showed a reduced level of PLA2 in the pancreas when compared with the naive mice, indicating the loss of PLA2 due to acinar injury^35^. In contrast, pancreatic PLA2 levels were comparable between healthy mice and mice injected with MΦ-NP(L&K), indicating the protection of normal acinar functions. Furthermore, the immune infiltration into the pancreatic parenchyma was examined by staining the pancreatic sections for CD45, a pan-leukocyte marker (Supplementary Fig. 13)^36^. As shown in Fig. 4j, a higher level of infiltrating leukocytes was found in CAE-AP mice compared to healthy naive mice. The level was also elevated in mice treated with MФ-NP, MФ-NP(mel), or MФ-NP(MJ). Compared to these groups, counts of CD45^+^ cells were significantly lower in mice treated with MΦ-NP(L&K), indicating a clear reduction of disease severity.

### Protection of mice with severe acute pancreatitis

**Fig. 5:**
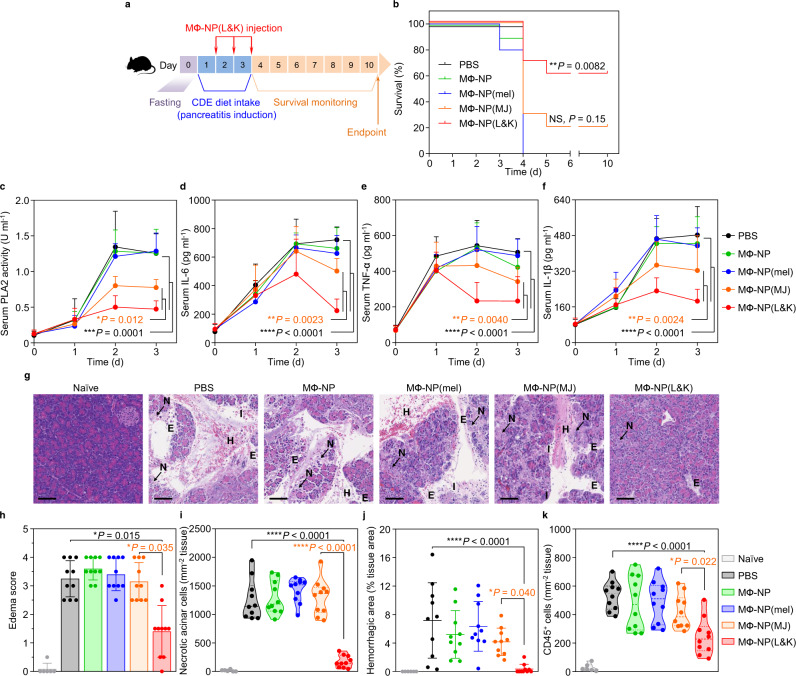
Therapeutic efficacy in a mouse model of severe acute pancreatitis. **a** The study protocol of lethal pancreatitis induction and treatment with MΦ-NP(L&K). **b** Survival rates of mice over 10 days after initial diet intake. NS not significant. **c** PLA2 activity profiles in serum of CDE-AP mice treated with different formulations. Concentration profiles of key inflammatory cytokines, including IL-6 (**d**), TNF-α (**e**), and IL-1β (**f**), of CDE-AP mice treated with different formulations. **g** Representative images of H&E staining on pancreas sections from CDE-AP mice collected on day 3 after initial diet intake. Scale bars, 100 μm. N acinar necrosis, E edema, I cell infiltration, H hemorrhage. **h** Edema score of H&E-stained pancreas sections from CDE-AP mice after receiving different nanoparticle treatments. **i** Quantification of necrotic cells in the H&E-stained pancreas sections from CDE-AP mice receiving different nanoparticle treatments. **j** Quantification of hemorrhagic area in the H&E-stained pancreas sections from of CDE-AP mice receiving different nanoparticle treatments. **k** Quantification of CD45^+^ cells in the anti-CD45-stained pancreas sections from CDE-AP mice treated with different formulations. For survival study, statistical analysis was performed on groups treated with MФ-MJ-NPs or MΦ-NP(L&K) in comparison with PBS-treated group using a two-tailed log-rank (Mantel–Cox) test. For serum PLA2 and cytokine profiles, statistical analysis was performed using repeated-measure one-way ANOVA with Dunnett’s post hoc analysis. Statistical difference in edema scores was analyzed by Kruskal–Wallis non-parametric test with Dunnett’s post hoc analysis. Necrotic cell counts, quantification of hemorrhagic area, and CD45^+^ cell counts were analyzed by using one-way ANOVA with Dunnett’s post hoc analysis. Data presented as mean ± s.d. *P ≤ 0.05, **P ≤ 0.01, ***P ≤ 0.001, ****P ≤ 0.0001. In the study, *n* = 10 mice in CDE-AP model. All mice were treated with the same batch of MΦ-NP(L&K). Source data are provided as a Source Data file for (**b**–**f**, **h**–**k**).

The protective effects of MΦ-NP(L&K) were further evaluated in a mouse model of severe AP (CDE-AP) induced by feeding mice with a choline-deficient diet supplemented with DL-ethionine. The diet intake period was positively correlated with the severity of AP (Supplementary Fig. 14)^37^. In the study, female CD-1 mice were placed on a CDE-diet for 3 days and each day MΦ-NP(L&K) were injected along with other control formulations for efficacy evaluation (Fig. 5a). A dosage of 80 mg/kg per injection was selected (Supplementary Fig. 15). As shown in Fig. 5b, all mice injected with PBS, MФ-NP, or MФ-NP(mel) died by day 4, indicating a lack of therapeutic efficacy from the control nanoparticles. Meanwhile, the survival rate of those injected with MФ-NP(MJ) was only 20%, suggesting ineffective protection if only MJ-33 was incorporated into the nanoparticles. In contrast, the survival rate of mice injected with MΦ-NP(L&K) increased significantly to 60%, demonstrating a clear benefit against AP development. As another evaluation of the efficacy at a systemic level, we monitored serum levels of PLA2, together with pro-inflammatory cytokines, including IL-6, TNF-α, and IL-1β, during days 0–3 of the study, a period prior to the occurrence of any death (Fig. 5c–f). Intake of CDE-diet drastically elevated serum PLA2 and cytokine levels in mice injected with PBS, which plateaued after day 2. On day 2 and day 3, serum PLA2 and cytokine levels in mice administered with MΦ-NP(L&K) remained significantly lower than those in all control groups. The results demonstrated the effective suppression of serum PLA2 and cytokines in CDE-AP, indicating a protective effect of MΦ-NP(L&K).

MΦ-NP(L&K) against severe AP was further evaluated by examining the histopathological changes in the pancreatic tissue during disease progression. In the study, mice were euthanized on day 3 of AP induction and the pancreatic tissues were collected for analyses. The pancreatic sections from naive mice displayed characteristics of a normal healthy pancreas including tight interlobular space and absence of acinar necrosis or immune infiltration (Fig. 5g). In contrast, the sections from CDE-AP mice injected with PBS showed marked parenchymal edema, pronounced acinar necrosis, and obvious signs of hemorrhage, which all are characteristic features of severe AP^37^. These features were also clearly observable in mice injected with MФ-NP, MФ-NP(mel), or MФ-NP(MJ), indicating a lack of therapeutic efficacy from these formulations. Comprehensive histopathological analyses revealed a significantly reduced edema score in mice injected with MΦ-NP(L&K), compared to mice receiving PBS or control formulations (Fig. 5h and Supplementary Figs. 16–21). In addition, counts of necrotic acinar cells were effectively reduced to basal levels in mice injected with MΦ-NP(L&K) (Fig. 5i), further confirming the protective effect of MΦ-NP(L&K) against severe AP. The hemorrhagic area in the pancreatic tissue was also measured and found to be reduced in mice treated with MΦ-NP(L&K) but not in those treated with PBS or control formulations (Fig. 5j). Pancreatic sections were also stained for CD45 for analyses of leukocyte infiltration (Fig. 5k and Supplementary Fig. 22). When compared to healthy naive mice, leukocyte infiltration in those injected with control formulations was significantly elevated but remained significantly lower for MΦ-NP(L&K)-treated mice. These results again confirmed the ability of MΦ-NP(L&K) in protecting animals against severe AP.

In summary, we exploited the susceptibility of macrophages to pancreatitis-associated PLA2 and designed a biomimetic nanoparticle with a ‘lure and kill” mechanism for safe and effective inhibition of PLA2. In this design, nanoparticles were made by wrapping with natural macrophage membranes doped with melittin and MJ-33 as PLA2 attractant and inhibitor, respectively. The modified macrophage membrane can eliminate the toxicity of free melittin and MJ-33 molecules while synergistically luring and killing PLA2. When tested in pancreatitis models, the resulting MΦ-NP(L&K) were shown to effectively inhibit PLA2 activity and PLA2-induced pancreatic injury. Their administration into mice with AP showed protective effects against AP-induced inflammation, tissue damage, and lethality.

The prominent anti-PLA2 activity of MΦ-NP(L&K) makes these biomimetic nanoparticles a promising drug candidate for the treatment of a wide range of PLA2-mediated diseases. Notably, in diseases such as cardiovascular disease, cancer, and autoimmune disorders, systemic PLA2 activity is positively correlated with disease severity and PLA2 inhibition is a desirable strategy^38–40^. In atherosclerosis, PLA2 is known to be responsible for remodeling low-density lipoprotein particles, promoting the aggregation of modified lipoproteins and the generation of foam cells^41^. PLA2 modulates cancer cell proliferation through the arachidonic acid metabolic pathway^39^. In autoimmune disorders such as rheumatoid arthritis, cytosolic PLA2 produces precursors for the synthesis of leukotriene and prostaglandin, which are known to contribute to disease progression^42^. Meanwhile, PLA2 isoforms show unique origins, substrate selectivity, and tissue distribution^12^. To address such complexity, nanoparticle formulations similar to MΦ-NP(L&K) can be made by coating with membranes from other cells sensitive to specific isoforms^43–45^. In addition, the cell membrane could be further modified with other unique compounds that either attract or inhibit PLA2^46,47^. In the study, we derived the membrane from macrophage cell lines. The possible immune mismatch between the source cells and the recipient animals might limit nanoparticle circulation lifetime. In this regard, source cells could be genetically modified prior to membrane collection to reduce such mismatch^48^. These features ensure MΦ-NP(L&K) design to be finely tailored to precisely target a specific PLA2 in the target disease. AP is a complex and evolving condition and generates multiple damaging factors yet to be understood^49–51^. While multiple animal models of AP have been developed, an effective treatment translatable to humans remains unavailable. Therefore, caution must be taken when envisioning the future development of AP treatment. With these challenges in place, we expect MΦ-NP(L&K) to be a useful tool toward developing therapies for AP and other related medical conditions.

## Methods

### Mice

Mice were housed in an animal facility at the University of California San Diego (UCSD) under federal, state, local, and National Institutes of Health (NIH) guidelines. Four-week-old or six-week-old CD-1 female mice were purchased from Envigo. Mice were maintained under standard housing with 12 h light/12 h dark cycle, ambient temperature, and normal humidity. All animal experiments were performed in accordance with NIH guidelines and approved by the Institutional Animal Care and Use Committee (IACUC) of UCSD.

### Macrophage culture

Mouse J774A.1 cell line (American Type Culture Collection, ATCC) was maintained in Dulbecco’s Modified Eagle Medium (DMEM, Invitrogen) supplemented with 10% FBS (Hyclone) and 1% penicillin-streptomycin (Invitrogen). Human THP-1 cell line (ATCC) was maintained in RPMI 1640 (Gibco) supplemented with 10% FBS and 1% penicillin-streptomycin. Cell cultures were maintained at 37 °C in a humidified incubator with 5% CO_2_ and regularly tested for mycoplasma contamination.

### Macrophage membrane derivation

The plasma membrane of macrophages was harvested by following a published protocol^27^. Briefly, J774A.1 cells were grown in T175 culture flasks to full confluency and detached with 3 mM ethylenediaminetetraacetic acid (EDTA, MilliporeSigma) in PBS. The cells were washed with 1× PBS three times (centrifugation at 800 × *g*) and suspended in a hypotonic lysing buffer containing 30 mM Tris-HCl (pH = 7.5), 225 mM D-mannitol, 75 mM sucrose, 0.2 mM ethylene glycol-bis(*β*-aminoethyl ether)-(*N,N,N*′*,N*′-tetraacetic acid) (EGTA), and protease and phosphatase inhibitor cocktails (all from MilliporeSigma). Cells were then disrupted using a Dounce homogenizer with a tight-fitting pestle (20 passes). The homogenized solution was centrifuged at 20,000 × *g* for 25 min at 4 °C. The pellet was discarded, and the supernatant was centrifuged again at 100,000 × *g* for 35 min at 4 °C. Following the centrifugation, membrane was collected as the pellet and washed twice with 0.2 mM EDTA in water. Membrane content was quantified by using a BCA protein assay kit (Thermo Fisher Scientific) in reference to a bovine serum albumin (BSA) standard. Approximately 1 × 10^8^ macrophages were able to yield 1 mg of membrane material (protein weight). The membrane was suspended with 0.2 mM EDTA to a protein concentration of 10 mg/ml and stored at −80 °C for subsequent studies. To collect membrane from THP-1 cells, the cells were grown in T175 suspension flasks (Olympus Plastics) to a density of 1 × 10^6^ cells/ml and washed three times in PBS (centrifugation at 800 × *g*). The plasma membrane was then harvested following the same procedure.

### Fabrication of MФ-NP

To synthesize nanoparticle cores, 0.4 ml poly(DL-lactic-*co*-glycolic acid) (50:50 PLGA, 0.67 dl/g, Lactel Absorbable Polymers) in acetone (20 mg/ml) was added dropwise into 1 ml water. The solution was placed below a vacuum aspirator until the acetone evaporated completely. To trace the nanoparticles, PLGA cores were prepared by co-dissolving PLGA with 0.1 wt% 1,1′-dioctadecyl-3,3,3′,3′-tetramethylindotricarbocyanine iodide (DiR, excitation = 750 nm/emission = 780 nm, ThermoFisher Scientific). For coating, the macrophage membrane was mixed with PLGA cores at a polymer-to-membrane protein weight ratio of 2:1. The mixture was then sonicated in a bath sonicator (Fisher Scientific FS30D) for 2 min, resulting in MФ-NP.

### Fabrication of MJ-incorporated macrophage membrane-coated nanoparticles

To incorporate MJ-33, macrophage membrane (4 mg/ml in water) was mixed with MJ-33 (0.04–1.28 mg/ml, corresponding to 1–32% membrane protein weight, dissolved in 100% ethanol, Cayman Chemicals) and sonicated with the bath sonicator for 30 s and incubated at 37 °C for 15 min. The same sonication-incubation cycle was repeated once. The suspension was centrifuged at 20,000 × *g* for 10 min and the MJ-33-loaded macrophage membrane was resuspended in water. To form nanoparticles, the MJ-33-loaded macrophage membrane was mixed with PLGA cores at a polymer-to-membrane protein weight ratio of 2:1. The mixture was then sonicated in the bath sonicator for 2 min for coating, resulting in MФ-NP(MJ). MФ-NP(MJ) were then washed twice with centrifugation. MJ-33 input into the macrophage membrane was optimized by measuring the inhibitory capacity of the resulting MФ-NP(MJ) against native bovine PLA2 (Creative Enzymes). Briefly, 160 μg/ml MФ-NP(MJ) with increasing MJ-33 input was mixed with native bovine PLA2 (600 U/l in PBS) and incubated at 37 °C for 1 h before the PLA2 activity was measured. Native bovine PLA2 (600 U/l in PBS) incubated at 37 °C for 1 h served as a positive control. PLA2 activity was measured by the EnzChek PLA2 activity assay kit (ThermoFisher Scientific) with a calibration curve measured with PLA2 standards of known enzymatic activity. PLA2 inhibition was calculated by subtracting the PLA2 activity of nanoparticle-PLA2 mixtures from that of the positive control sample. MJ-33 input that resulted in the maximum PLA2 inhibition (16% of membrane protein weight) was used for subsequent studies.

### Fabrication of lure and kill macrophage membrane-coated nanoparticles

To fabricate MΦ-NP(L&K), 2 mg/ml MФ-NP(MJ) was incubated with melittin (5–160 μg/ml, corresponding to 0.25–8% membrane protein weight, MilliporeSigma) at 37 °C for 30 min. MΦ-NP(L&K) were then washed twice with centrifugation. Melittin input into MФ-NP(MJ) was optimized by measuring the inhibitory capacity of the resulting MΦ-NP(L&K) against native bovine PLA2. Briefly, MΦ-NP(L&K) (160 μg/ml) with increasing melittin input was mixed with 600 U/l native bovine PLA2 and incubated at 37 °C for 1 h. PLA2 activity was measured to calculate PLA2 inhibition by MΦ-NP(L&K). PLA2 inhibition was calculated using the same method as mentioned above. The melittin input that resulted in the maximum PLA2 inhibition (4% of membrane protein weight) was used to prepare MΦ-NP(L&K) for all subsequent studies.

### Quantitative analysis of MJ-33 loading yield

Freshly prepared MФ-NP(MJ) or MΦ-NP(L&K) (30 μL, 2 mg/mL) were centrifuged at 25,000 × *g* for 10 min and resuspended in 100 μL ultrapure water. To extract cell membrane lipids, 200 μL methanol was added to the nanoparticle suspensions and the samples were vigorously vortexed for 30 s. Then 400 μL chloroform was added and the mixture was shaken at room temperature for 30 min. After that, the samples were centrifuged at 2500 × *g* for 5 min. The organic solvent at the bottom was collected, dried under nitrogen flow, and reconstituted in 100 μL methanol for HR-ESI-MS analysis. MФ-NP (30 μL, 2 mg/mL) were processed by following the same procedure and served as a control sample. MJ-33 content in the nanoparticle samples was derived from the relative abundance value (m/z = 491.3) from the nanoparticle sample using the MJ-33 calibration curve. MJ-33 loading yield was calculated as the weight percentage of MJ-33 in the total membrane protein. Results were fitted by using linear regression function in Graphpad Prism 8.

### Quantitative analysis of melittin loading yield

Melittin loading into MΦ-NP(L&K) was derived by measuring the hemolytic activity of melittin solution before and after the incubation with the MФ-NP(MJ). To quantify melittin loading into MΦ-NP(L&K), MФ-NP(MJ) (final concentration 2 mg/ml) were mixed with melittin (final concentration 10–160 μg/mL) and incubated at 37 °C for 30 min. The mixture was diluted 10× in PBS and added to washed mouse RBCs (5 v/v%) in PBS and incubated at 37 °C for 30 min. Afterwards, the RBC suspension was centrifuged at 16,100 × *g* for 5 min. Then 20 μL supernatant was added into 80 μL PBS in 96-well plates. Hemoglobin absorption was measured at 540 nm. RBCs (5 v/v%) in PBS were disrupted with sonication and centrifuged at 16,100 × *g* for 5 min. The supernatant of sonicated RBCs (5 v/v%) in PBS was taken as 100% hemolysis, and the supernatant of intact RBCs (5 v/v%) in PBS was taken as 0% hemolysis. Hemolysis (%) was defined as (A_sample_ − A_intact_)/(A_sonicated_ − A_intact_) × 100%. The remaining melittin concentration after incubation with the nanoparticles was derived from the calibration curve for melittin-induced hemolysis. Melittin loading was calculated as the weight percentage of melittin in total membrane protein. Results were fitted by using non-linear fitting in Graphpad Prism 8.

### Physicochemical properties, morphology, and colloidal stability

MΦ-NP(L&K) were measured for hydrodynamic size and surface zeta potential with dynamic light scattering (DLS, ZEN 3600 Zetasizer, Malvern). To examine the intra-particle structure, nanoparticles were stained with uranyl acetate (0.2 wt%) and visualized with transmission electron microscopy (TEM, FEI 200 kV Sphera). To study the long-term colloidal stability, MΦ-NP(L&K) suspensions were adjusted to 1× PBS or 0.5× FBS, at a final protein concentration of 0.5 mg/ml. Samples were stored at 37 °C and nanoparticle hydrodynamic size was measured and monitored once a day for 7 consecutive days.

### Cytotoxicity evaluation

J774A.1 macrophages were seeded in a 96-well tissue culture plate at a density of 5 × 10^4^ cells/ml and cultured overnight. Cells were then added with various concentrations of free MJ-33 or MΦ-NP(L&K) with equivalent MJ-33 content. Cells were then cultured for 24 h. Cell viability was assessed by using a CellTiter Cell Proliferation assay (Promega) based on the manufacturer’s instruction.

### Mouse model of mild acute pancreatitis

A mild acute pancreatitis (CAE-AP) mouse model was established by following a published protocol^36^. Briefly, 6-week-old CD-1 female mice were fasted 12 h before pancreatitis induction and given ad libitum access to water. Mice received intraperitoneal injections of caerulein (VWR) at 50 μg/kg hourly for 8 h to induce AP.

### Inhibition of PLA2 in serum samples from CAE-AP mice and patients

To collect serum from CAE-AP mice, the whole blood of mice was collected with submandibular bleeding into microtubes and allowed to clot at room temperature for 30 min. Samples were then centrifuged at 2000 × *g* for 6 min to collect serum from the supernatant. The serum was then lyophilized and reconstituted to 1/10 of its volume (equivalent to a 10× serum concentration). Serum samples collected from human AP patients were purchased from BioreclamationIVT. Aliquots of samples were stored at −80 °C and used within 2 weeks after the collection. In the study, mouse serum samples (0.1× final concentration) were mixed with MΦ-NP(L&K) or control formulations (final concentrations ranging from 0.625 to 160 μg/ml). Samples were incubated at 37 °C for 1 h before PLA2 activity was measured. For studies with human serum samples, MΦ-NP(L&K) made from the membrane of THP-1 cells were used. In the study, patient serum samples (0.1× final concentration) were mixed with MΦ-NP(L&K) or control formulations (final concentrations ranging from 0.016 to 4 mg/ml). Samples were incubated at 37 °C for 1 h before the PLA2 activity was measured. IC_50_ values were derived from the variable slope model using Graphpad Prism 8.

### Pharmacokinetics and biodistribution assessments

To characterize the pharmacokinetics and biodistribution of MΦ-NP(L&K) in vivo, MΦ-NP(L&K) were prepared from DiR-loaded PLGA cores. To study pharmacokinetics, 200 μl of 3 mg/ml fluorescently labeled nanoparticles were administered intravenously into 4-week-old CD-1 female mouse. Blood samples were collected by submandibular bleeding at 3 min, 30 min, 1 h, 3 h, 7 h, 24 h, 48 h, and 72 h. Samples were then diluted 10× with PBS and the fluorescence intensity was measured (Tecan Infinite M200 multiplate reader). Pharmacokinetic parameters were calculated by fitting the curve with a two-compartment model. For the biodistribution study, 200 μl of 3 mg/ml fluorescently labeled nanoparticles were administered intravenously into 4-week-old CD-1 female mice. At 24, 48, and 72 h after the injection, organs, including the liver, kidneys, spleen, lungs, heart, blood, and pancreas, were collected from a randomly grouped subset of mice. Organs were weighed and then homogenized in PBS with a Mini-Beedbeater-16 (Biospec Products) for fluorescence measurement.

### Characterization of in vivo toxicity

To evaluate the toxicity, MΦ-NP(L&K) were injected intravenously (80 mg/kg) through the tail vein into 4-week-old CD-1 female mice daily for 4 days. At 24 h after the last injection, ~250 μl of whole blood was collected into Eppendorf tubes and allowed to coagulate. Then the samples were centrifuged at 2000 × *g* for 6 min to collect serum for the comprehensive metabolic panel analysis. Meanwhile, 100 μl of whole blood was collected into an EDTA-coated microtube for the complete blood count. All blood samples were tested at the UCSD Animal Care Program Diagnostic Services Laboratory. Immediately after blood collection, mice were euthanized to collect major organs, including the liver, kidneys, spleen, lungs, heart, and pancreas. Organs were fixed in 10% formalin (Fisher Scientific), sectioned, and stained with hematoxylin and eosin (H&E) for histological analysis. Histology slides were imaged with a Hamamatsu NanoZoomer 2.0-HT slide scanning system.

### Assessment of NF-κB nuclear translocation in macrophages

J774A.1 macrophages were seeded in Celltreat tissue culture-treated dishes with a glass bottom (Neta Scientific) at a density of 5 × 10^4^ cells/ml and cultured for 24 h. Serum from CAE-AP mice was reconstituted with the culture medium to a final PLA2 activity of 600 U/l. Then, MΦ-NP(L&K) or control nanoparticles were mixed with the culture medium to reach a final concentration of 400 μg/ml (membrane protein). The final volume in each culture dish was adjusted to 500 μl using the culture medium. Cells were incubated for 2 h at 37 °C and then washed with PBS 3 times. Washed cells were fixed with 10% formalin for 15 min, then permeabilized and blocked with a PBS solution containing 0.4% Triton X-100 (Fisher Scientific) and 2% BSA for 30 min at room temperature. Intracellular NF-κB was probed with anti-mouse NF-κB p65 antibody (clone A-8, 1:200 dilution in PBS, Santa Cruz Biotechnology) for 1 h at room temperature. Cells were then washed twice with PBS and FITC-anti-mouse IgG (1:500 dilution in PBS, Biolegend) was added to the cells for 20 min at room temperature. Cells were washed twice and stained with 0.1 μg/ml Hoechst 33342 (ThermoFisher Scientific) for 20 min at room temperature. Fluorescence was visualized using a Leica SP8 confocal microscope. Quantification of NF-κB nuclear translocation was performed by following a published protocol using ImageJ^52^. Briefly, the Hoechst-positive area was used to define the nuclear region of interest (ROI) for each nucleus. NF-κB-positive area encompassing each nuclear ROI was defined as the ROI for a whole cell. NF-κB signal within a nucleus was measured by the total fluorescence intensity within the nuclear ROI. NF-κB signal within a whole cell was measured by the total fluorescence intensity within the ROI of a whole cell. Nuclear translocation (%) was defined as Fluo_nuclei_/Fluo_whole cell_ × 100 %. One hundred cells were measured individually for each group. Nuclear translocation histograms were fitted with Gaussian non-linear regression by using GraphPad Prism 8.

### Measurement of macrophage cytokine production

J774A.1 macrophages were seeded in 96-well tissue culture plates at a density of 8 × 10^4^ cells/ml and cultured overnight. Serum from CAE-AP mice was reconstituted with the culture medium to a final PLA2 activity of 600 U/l. Afterward, MΦ-NP(L&K) or control nanoparticles were mixed with the culture medium to reach final concentrations ranging from 3.125 to 400 μg/ml in membrane protein concentration. The final volume in each well was adjusted to 100 μl using the culture medium. Cells were incubated for 6 h at 37 °C and the culture medium was collected. Concentrations of inflammatory cytokines IL-6, TNF-α, and IL-1β in the culture medium was quantified by using the corresponding ELISA kits (Biolegend).

### Isolation of pancreatic acini

Six-week-old healthy CD-1 mice were euthanized, and the pancreatic tissues were collected. Minced pancreatic tissues were dispersed in DMEM with Nutrient Mixture F-12 (DMEM/F12, Gibco) supplemented with 1.0 U/ml collagenase D from *Clostridium histolyticum* and 0.25 mg/ml trypsin inhibitor from soybean (both from Roche Diagnostics). The pancreatic tissues were vigorously shaken at 200 rpm for 2 h at 37 °C. Digested tissue was filtered by using a cell strainer (100 μm pore size, Corning). Pancreatic acini retained by the cell strainer were transferred to a 24-well tissue culture plate and cultured overnight in DMEM/F12 medium supplemented with 5% FBS and 1% penicillin-streptomycin. Non-adherent pancreatic acini were then transferred into a new 48-well tissue culture plate and used immediately, while adherent contaminant cells were discarded.

### Pancreatic acinar cell injury assay

Pancreatic acini were suspended in DMEM/F12 medium at a density of 800 acini/ml, and 100 μl pancreatic acini suspension was added to each well of a 48-well tissue culture plate. Serum from CAE-AP mice was first added to the acini suspension to a final PLA2 activity of 600 U/l. MΦ-NP(L&K) or control nanoparticles were then added to the acini suspension to reach final concentrations ranging from 3.125 to 400 μg/ml in membrane protein concentration. The final volume in each culture dish was adjusted to 200 μl using the culture medium. Pancreatic acini were cultured for 24 h and washed three times with PBS. Cell viability was measured by using a CellTiter Cell Proliferation assay (Promega). To study the cell death pathway of PLA2-induced acinar cell injury, serum from CAE-AP mice was added to the acini suspension (800 acini/ml in 100 μl culture medium) to a final PLA2 activity of 600 U/l. Then, MΦ-NP(L&K) or control nanoparticles were mixed with the acini suspension to reach a final concentration of 400 μg/ml in membrane protein concentration. The final volume in each well was adjusted to 200 μl using the culture medium. The mixtures were incubated for 24 h at 37 °C and then washed with PBS for 3 times. PACs were then stained with PE-labeled annexin-V (1:100 dilution, Biolegend) and propidium iodide (1:5000 dilution, Biolegend), and analysed with a Becton Dickinson FACSCanto-II flow cytometer. Results were analysed using FlowJo software.

### Choline-deficient diet and DL-ethionine induced acute pancreatitis mouse model

The mouse model was established by following a published protocol^53^. Briefly, 4-week-old CD-1 female mice were fasted on day 0 and then fed with choline-deficient and DL-ethionine diet (CDE-diet, MP Biomedicals) from days 1 to 3, followed by normal laboratory diet on day 4 to induce lethal AP. Survival was monitored for 10 days. Throughout the CDE-diet induced acute pancreatitis (CDE-AP) studies, mice had access to water ad libitum.

### Protocols of efficacy study in CAE-AP and CDE-AP

In CAE-AP, 2 h after the initial caerulein administration, 200 μl of MΦ-NP(L&K) (40 mg/kg) was administered intravenously through the tail vein. MФ-NP (40 mg/kg), MФ-NP(mel) (40 mg/kg), MФ-NP(MJ) (40 mg/kg), or 200 μl sterile PBS was administered intravenously to mice at the same time as controls. CAE-AP mouse whole blood was collected at 0, 2, 4, 8, 24 h after initial caerulein injection (~80 μl whole blood per mouse per timepoint) with submandibular bleeding into microtubes and allowed to clot at room temperature for 30 min. Samples were then centrifuged at 2000 × *g* for 6 min to collect serum from the supernatant. Serum samples were immediately frozen at −20 °C and analysed for PLA2 activity and cytokine concentration within 24 h. All mice were euthanized at 24 h after initial caerulein injection to collect pancreatic tissues for measurement of pancreatic tissue PLA2 activity and histological analyses. In CDE-AP studies, 200 μl of MΦ-NP(L&K) (80 mg/kg) was administered intravenously through the tail vein on days 1, 2, and 3 of the studies. MФ-NP (80 mg/kg), MФ-NP(mel) (80 mg/kg), MФ-NP(MJ) (80 mg/kg), or 200 μl sterile PBS was administered intravenously to mice at the same time as controls. The whole blood of CDE-AP mice was collected on days 0, 1, 2, and 3 (~80 μl whole blood per mouse per timepoint). CDE-AP mouse serum samples were derived and stored following the aforementioned procedures. Pancreatic tissues were collected on day 3 of the CDE-AP studies for histological analyses.

### Measurement of PLA2 activity and cytokine levels in serum

Serum samples from CAE-AP mice and CDE-AP mice were diluted 5× in PBS and serum PLA2 activity was quantified by using the EnzChek PLA2 assay kit. Serum cytokine concentrations were measured by using mouse IL-6, mouse TNF-α, and mouse IL-1β ELISA kits. To measure PLA2 activity in CAE-AP mouse pancreatic tissues, freshly collected mouse pancreas samples were homogenized with a Mini-Beedbeater-16 (Biospec Products). The homogenate was immediately diluted 10× in PBS and analysed for PLA2 activity. PLA2 activity results were normalized to the weight of pancreatic tissues.

### Histological analysis of pancreatic tissues

For histological analyses, CAE-AP mouse and CDE-AP mouse pancreatic tissues were fixed, sectioned, and stained with H&E. H&E-stained sections were visualized by a Hamamatsu NanoZoomer 2.0-HT slide scanning system. Histopathological features including edema, necrotic acinar cells, hemorrhage, and CD45^+^ cell infiltration were studied. To analyze parenchymal edema in the pancreatic tissue in CAE-AP and CDE-AP models, a representative 0.45 × 0.45 mm area was selected from each H&E-stained pancreatic section. The selected area was scored by a blinded subject who was unaware of the type of treatment administered to the animals. Score 0 = no edema; 0.5 = focal expansion of interlobular space; 1 = diffuse expansion of interlobular space; 1.5 = focal expansion of intralobular space; 2 = diffuse expansion of intralobular space; 2.5 = focal expansion of inter-acinar space; 3 = diffuse expansion of inter-acinar space; 3.5 = focal expansion of intercellular space; 4 = diffuse expansion of intercellular space. To quantify the necrotic cells in the pancreatic parenchyma in CAE-AP and CDE-AP models, five 0.15 × 0.15 mm areas were randomly selected from each H&E-stained pancreatic section. Necrotic cells in each area were counted. To study the extent of hemorrhage in the CDE-AP model, a representative 0.6 × 0.6 mm area was selected from each pancreatic section. The hemorrhagic area was defined as the area containing extravasated erythrocytes and quantified using Adobe Photoshop. Counts of necrotic acinar cells and hemorrhagic area results were normalized to the area occupied by the pancreatic tissue. To study CD45^+^ cell infiltration into the pancreas, pancreatic tissues were sectioned and stained with a rabbit anti-mouse CD45 antibody (Abcam, 1:200 dilution). Biotinylated anti-rabbit IgG (Abcam, 1:500 dilution) was used as the secondary antibody for chromagen development. Sections were counter-stained with haematoxylin to visualize cell nuclei and were scanned by a Hamamatsu NanoZoomer 2.0-HT slide scanning system. A 0.15 × 0.15 mm area in each anti-CD45-stained pancreatic section was selected to quantify CD45^+^ cell infiltration using ImageJ. Briefly, RGB images of anti-CD45-stained pancreatic sections were converted to 16-bit grayscale images. A standard threshold was applied to exclude CD45^-^ cells. The remaining CD45^+^ cells were counted by using ImageJ Particle Analyzer. Counts of CD45^+^ cells were normalized to the area occupied by the pancreatic tissue.

### Statistical analysis

Statistical analyses of serum biomarkers were performed using a repeated-measure one-way ANOVA with Dunnett’s post hoc analysis. Statistical difference in edema scores was analyzed by Kruskal–Wallis non-parametric test with Dunnett’s post hoc analysis. Necrotic cell counts, tissue PLA2 activity, and CD45^+^ cell counts were analyzed by using one-way ANOVA with Dunnett’s post hoc analysis. Animal survival data were analysed by using the log-rank (Mantel–Cox) test. In the CAE-AP study, *n* = 10 mice in all groups. In CDE-AP studies, *n* = 10 mice in all groups. Replicates represent different mice subjected to the same treatment.

### Reporting summary

Further information on research design is available in the Nature Research Reporting Summary linked to this article.

## Supplementary information

Supplementary Information

Reporting Summary

## Data Availability

All data are available within the article, Supplementary Information or available from the corresponding authors upon reasonable request. Source data are provided with this paper.

## Source data

Source Data
