# Supplementary material for: Lure-and-kill macrophage nanoparticles alleviate the severity of experimental acute pancreatitis
Source: Nat Commun. 2021 Jul 6;12:4136. doi: 10.1038/s41467-021-24447-4 (PMC8260623; doi:10.1038/s41467-021-24447-4)
Supplement: Supplementary file 1 — Supplementary Information [file 41467_2021_24447_MOESM1_ESM.pdf]

Lure-and-kill macrophage nanoparticles alleviate the severity of experimental acute pancreatitis

Qiangzhe Zhang, Julia Zhou, Jiarong Zhou, Ronnie H. Fang, Weiwei Gao, and Liangfang Zhang*

Department of Nanoengineering, Chemical Engineering Program, Moores Cancer Center, University of California San Diego, La Jolla, CA 92093, USA

* Correspondence author: zhang@ucsd.edu

Supplementary Information

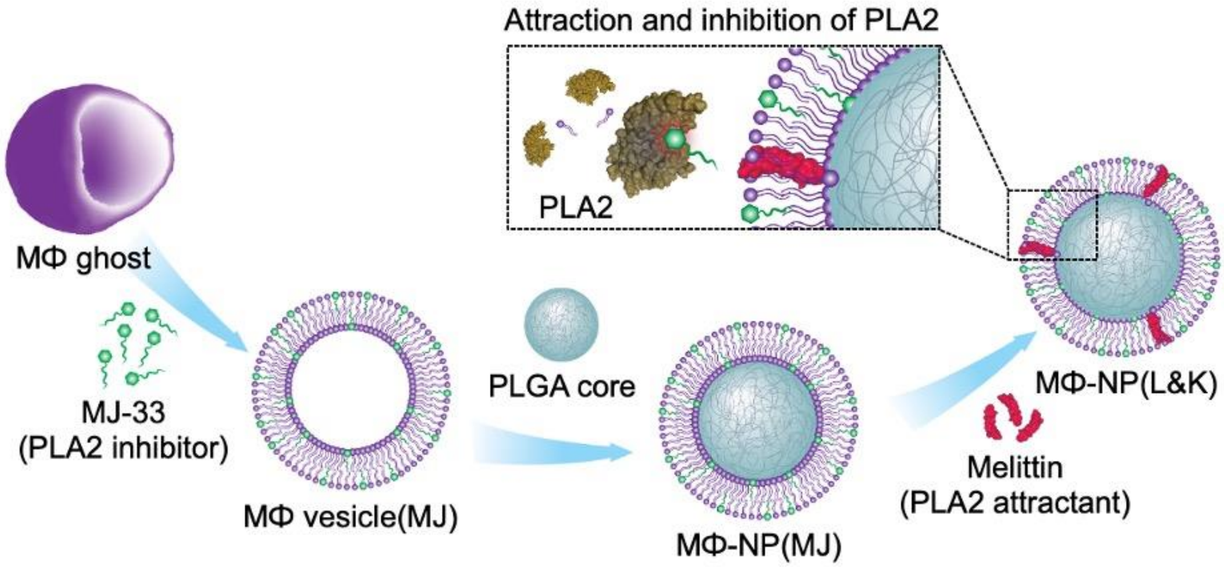

Supplementary Fig. 1. Schematic illustration of the formulation of MΦ-NP(L&K) and its use for PLA2 inhibition. To prepare MΦ-NP(L&K), phosphoric acid, mono[1-[(hexadecyloxy)methyl]-2-(2,2,2-trifluoroethoxy)ethyl] monomethyl ester, monolithium salt (MJ-33, a PLA2 inhibitor) and melittin (a PLA2 attractant) are incorporated into the membrane of macrophage (MΦ), which is then coated onto a polymeric core. MΦ-NP(L&K) acts as decoy to react with PLA2 and subsequently inhibits the enzymatic activity.

A calibration curve correlating MJ-33 concentration with relative abundance signal from HR-ESI-MS was first established. Pure MJ-33 monolithium salt (Mw = 498.5 g mol^{-1}) was dissolved in methanol to reach final concentrations of 0.001 – 0.1 mg ml^{-1}. Samples were analyzed by using HR-ESI-MS. Characteristic molecular ion peak was identified at m/z = 491.3, in agreement with the theoretical molecular weight of MJ-33. Relative abundance signal from samples containing different MJ-33 concentrations were normalized to the signal at m/z = 491.3 of 0.1 mg ml^{-1} MJ-33 to construct a calibration curve. To study MJ-33 incorporation into the nanoparticle, freshly prepared MΦ-NP(MJ) or MΦ-NP(L&K) were processed as described in the methods. MJ-33 content in the nanoparticle samples were derived from the relative abundance value (m/z = 491.3) from the nanoparticle sample using the MJ-33 calibration curve. MJ-33 loading yield was calculated as the weight percentage of MJ-33 in total membrane protein. Results were fitted by using linear regression function in Graphpad Prism 8.

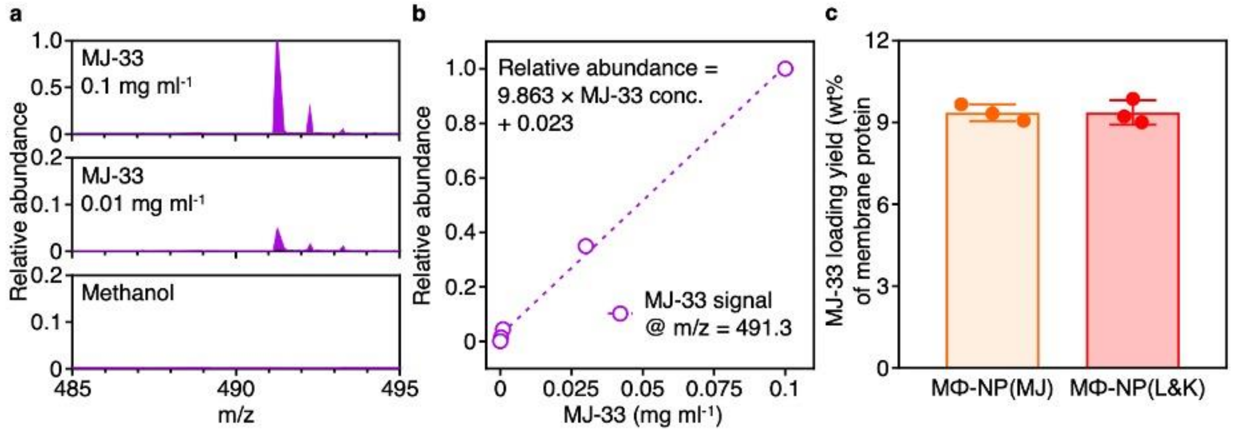

Supplementary Fig. 2. Analysis of MJ-33 incorporation into MΦ-NP(L&K) using HR-ESI-MS. **a**, Characteristic molecular ion peaks of MJ-33 (0.1 and 0.01 mg ml^{-1}) and methanol (solvent). **b**, Calibration curve correlating MJ-33 concentration with relative abundance signal from MS analysis. **c**, MJ-33 loading yield (9.4 ± 0.4 wt% of membrane protein) in MΦ-NP(MJ) and MΦ-NP(L&K) samples (n = 3 independent experiments using the same batch of MΦ-NP(MJ) and MΦ-NP(L&K), data presented as mean ± s.d.). Source data are provided as a Source Data file for Supplementary Fig. 2**a**-**c**.

A melittin calibration curve was constructed by measuring hemolytic activity of melittin. Briefly, various concentrations of melittin in PBS was added to 5 v/v% washed mouse RBC in PBS and incubated at 37 °C for 30 min. Afterwards, the RBC suspension was centrifuged at 16,100 ×*g* for 5 min. Then 20 μL supernatant was added into 80 μL PBS in 96-well plates. Hemoglobin absorption was measured at 540 nm. RBCs (5 v/v%) in PBS were disrupted with sonication and centrifuged at 16,100 ×*g* for 5 min. The supernatant of sonicated RBCs (5 v/v%) in PBS was taken as 100% hemolysis, and the supernatant of intact RBCs (5 v/v%) in PBS was taken as 0% hemolysis. Hemolysis (%) was defined as $(A_{sample} – A_{intact}) / (A_{sonicated} – A_{intact}) \times 100\%$. Remaining melittin concentration after incubation with the nanoparticles was derived from this calibration curve of melittin-induced hemolysis.

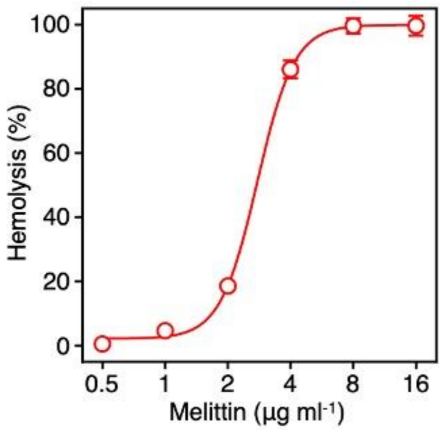

Supplementary Fig. 3. Construction of melittin-induced hemolysis calibration curve. All data points represent mean ± s.d. (n = 3 independent experiments using the same batch of melittin). Source data are provided as a Source Data file for Supplementary Fig. 3.

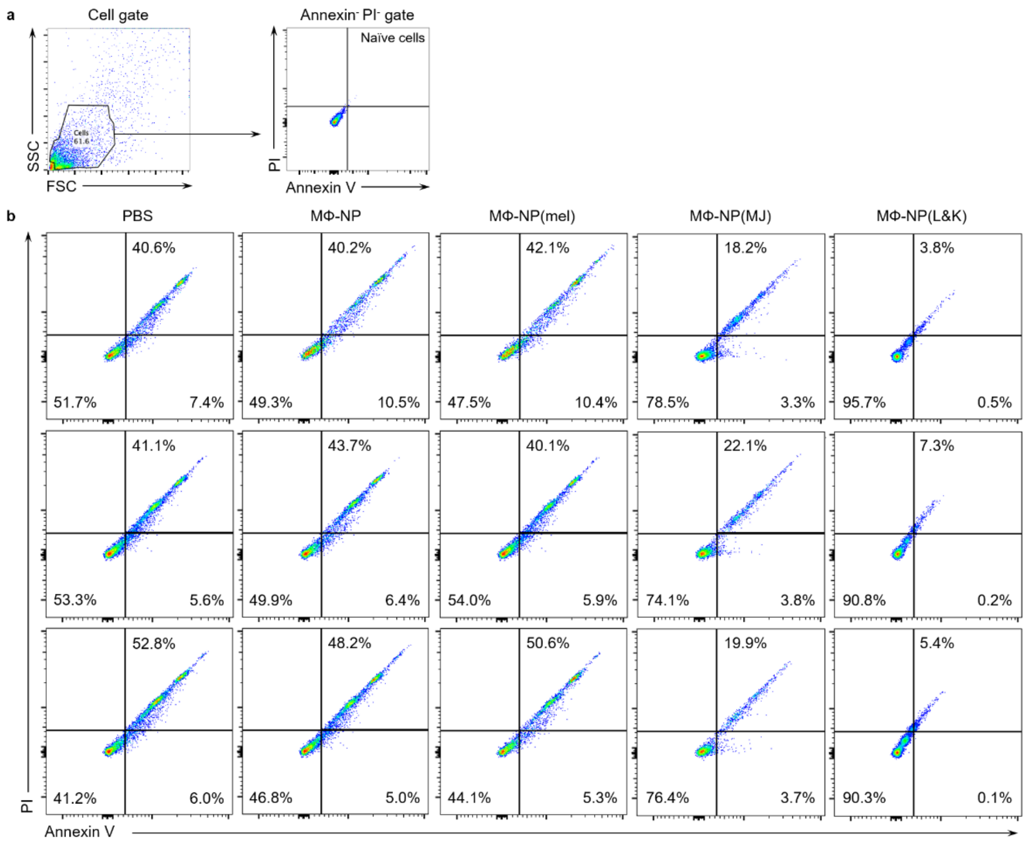

Supplementary Fig. 4. Flow cytometric analysis of the effect of MΦ-NP(L&K) treatment on PLA2-induced pancreatic acinar cell injury. Concentrated serum from CAE-AP mice (final PLA2 activity 600 U l^{-1}) was added to pancreatic acinar cells. MΦ-NP(L&K) (final concentration 400 μg ml^{-1}) or control formulations (MΦ-NP, MΦ-NP(mel), or MΦ-NP(MJ)) were mixed with the culture medium immediately after the addition of concentrated CAE-AP mouse serum. After 24 h incubation, pancreatic acinar cells were stained with PE-labeled annexin-V and propidium iodide (both from Biolegend) and analyzed by flow cytometry. **a**, Gating strategy used to identify necrotic cell population (annexin$^+$ PI$^+$) and apoptotic cell population (annexin$^+$ PI$^-$) in acinar cells. Naïve cells were used to establish (annexin$^-$ PI$^-$) gates. **b**, Scatter plots of CAE-AP serum-stimulated cell populations treated with MΦ-NP(L&K) or control formulations. The addition of MΦ-NP(L&K) inhibited the cytotoxic effect of PLA2 on pancreatic acinar cells, by reducing the necrotic cell population (annexin$^+$ PI$^+$) and apoptotic cell population (annexin$^+$ PI$^-$).

To study the effect of escalating doses of MΦ-NP(L&K) against CAE-AP, 2 h after the initial caerulein administration, 200 µl of MΦ-NP(L&K) (10 or 40 mg kg^{-1}) was administered intravenously through the tail vein. Sterile PBS (200 µl) was administered intravenously to mice at the same time as controls. CAE-AP mouse whole blood was collected at 4 h after initial caerulein injection (~100 µl whole blood per mouse) with submandibular bleeding into microtubes and allowed to clot at room temperature for 30 min. Samples were then centrifuged at 2,000 ×*g* for 6 min to collect serum from the supernatant. Serum samples were immediately frozen at -20°C and analysed for PLA2 activity and cytokine concentration within 24 h.

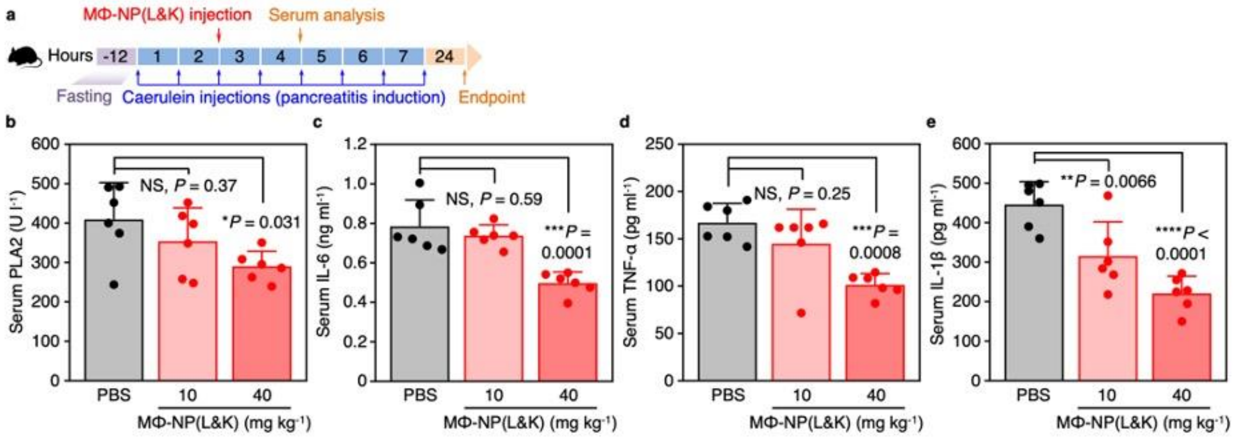

Supplementary Fig. 5. Effect of MΦ-NP(L&K) dosage on alleviating PLA2-induced inflammatory response in a mouse model of CAE-AP. **a**, The study protocol of pancreatitis induction and treatment with MΦ-NP(L&K) (10 or 40 mg kg^{-1}). **b**, PLA2 activity at 4 h after initial caerulein administration in serum of CAE-AP mice treated with different doses of MΦ-NP(L&K). **c-e**, Concentrations of key inflammatory cytokines including IL-6 (**c**), TNF-α (**d**), and IL-1β (**e**) at 4 h after initial caerulein administration in the serum of CAE-AP mice treated with different doses of MΦ-NP(L&K). For all datasets, statistical analysis was performed using one-way ANOVA with Dunnett's post hoc analysis. NS, not significant. Data presented as mean ± s.d. In all datasets, n = 6 mice treated with the same batch of MΦ-NP(L&K). Source data are provided as a Source Data file for Supplementary Fig. 5**b-e**.

Pancreatic tissues from CAE-AP mice and CDE-AP mice were fixed, sectioned, stained with H&E and anti-CD45 antibody, and visualized by a Hamamatsu NanoZoomer 2.0-HT slide scanning system. Histopathological features including parenchymal edema, necrotic acinar cells, hemorrhage, and $CD45^+$ cell infiltration were studied. To analyze parenchymal edema in the pancreatic tissue in CAE-AP and CDE-AP models, a representative 0.45 mm × 0.45 mm area was selected from each H&E-stained pancreatic section. The selected area was scored by a blinded subject who was unaware of the type of treatment administered to the animals. To quantify the necrotic acinar cells in the pancreatic parenchyma of CAE-AP and CDE-AP mice, five 0.15 mm × 0.15 mm areas were randomly selected from each H&E-stained pancreatic section. Necrotic acinar cells in each area were counted. To study the extent of hemorrhage in the CDE-AP model, a representative 0.6 mm × 0.6 mm area was selected from each pancreatic section. The hemorrhagic area was defined as the area containing extravasated erythrocytes and quantified using Adobe Photoshop. Lastly, a 0.15 mm × 0.15 mm area in each anti-CD45-stained pancreatic section was selected to quantify the number of $CD45^+$ cells as described before. Counts of necrotic acinar cells, hemorrhagic area, and counts of $CD45^+$ cells were normalized to the area occupied by the pancreatic tissue.

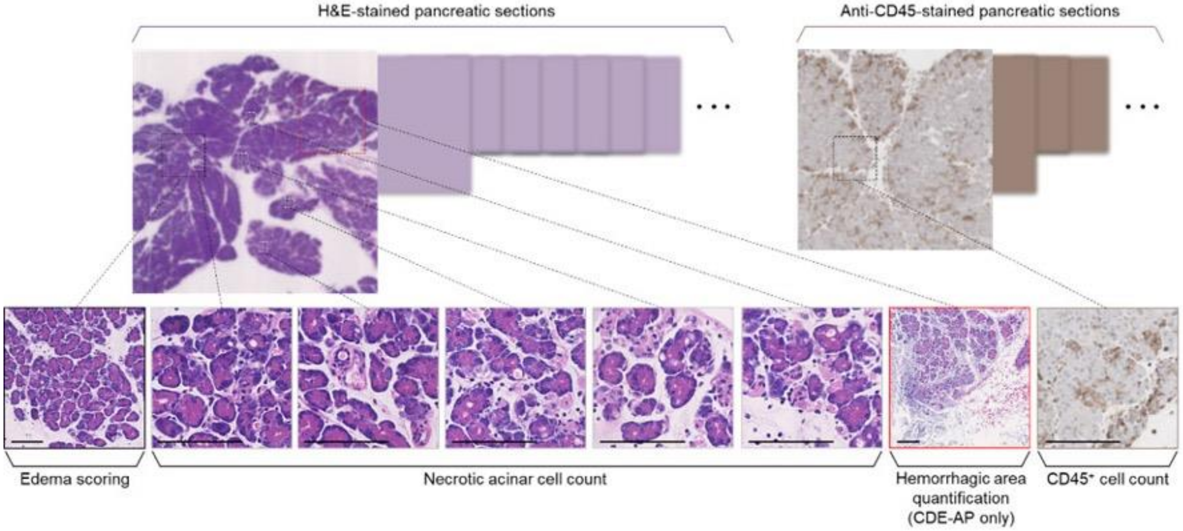

Supplementary Fig. 6. Workflow for histopathological analysis of pancreatic sections from CAE-AP and CDE-AP mice.

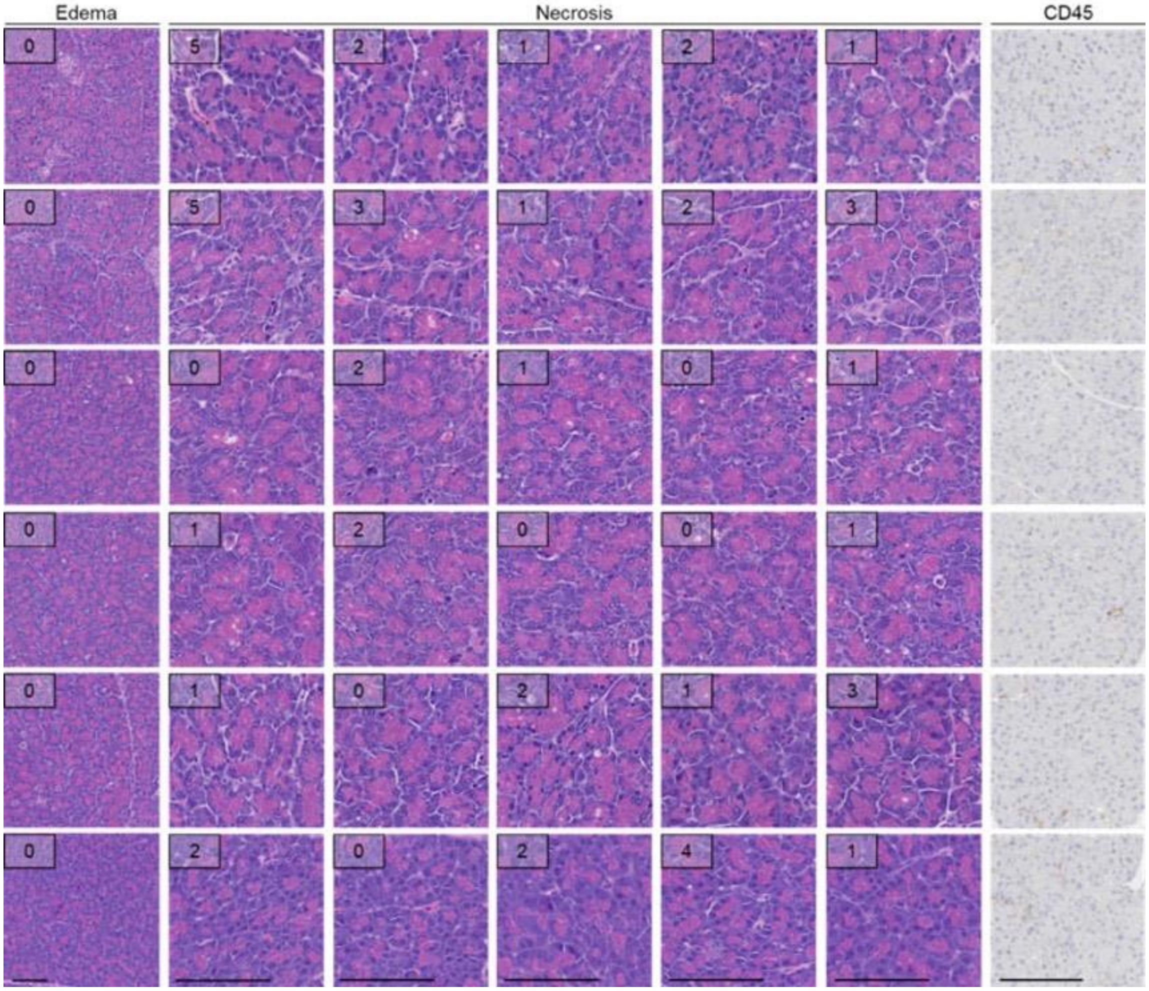

Supplementary Fig. 7. Histopathological analysis of pancreatic sections from healthy naïve mice in the CAE-AP study. Edema score and counts of necrotic acinar cells were labeled on each image. Each row of images included selected areas from the same pancreas. Scale bars = 100 μm. In the study, n = 6 mice for healthy control group. The study was repeated twice independently with similar results.

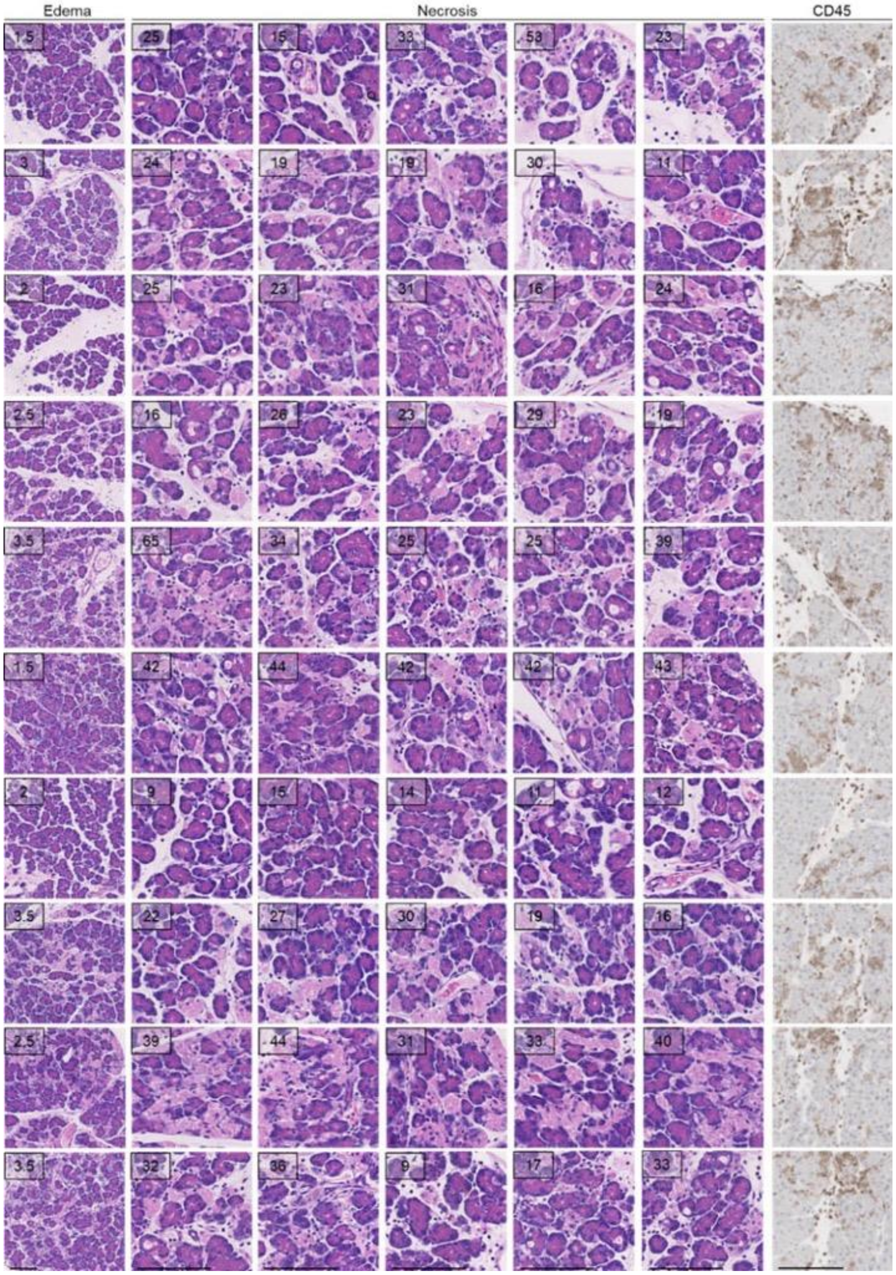

Supplementary Fig. 8. Histopathological analysis of pancreatic sections from PBS-treated CAE-AP mice. Edema score and counts of necrotic acinar cells were labeled on each image. Each row of images included selected areas from the same pancreas. Scale bars = 100 μm. In the study, n = 10 mice for PBS treatment group. The study was repeated twice independently with similar results.

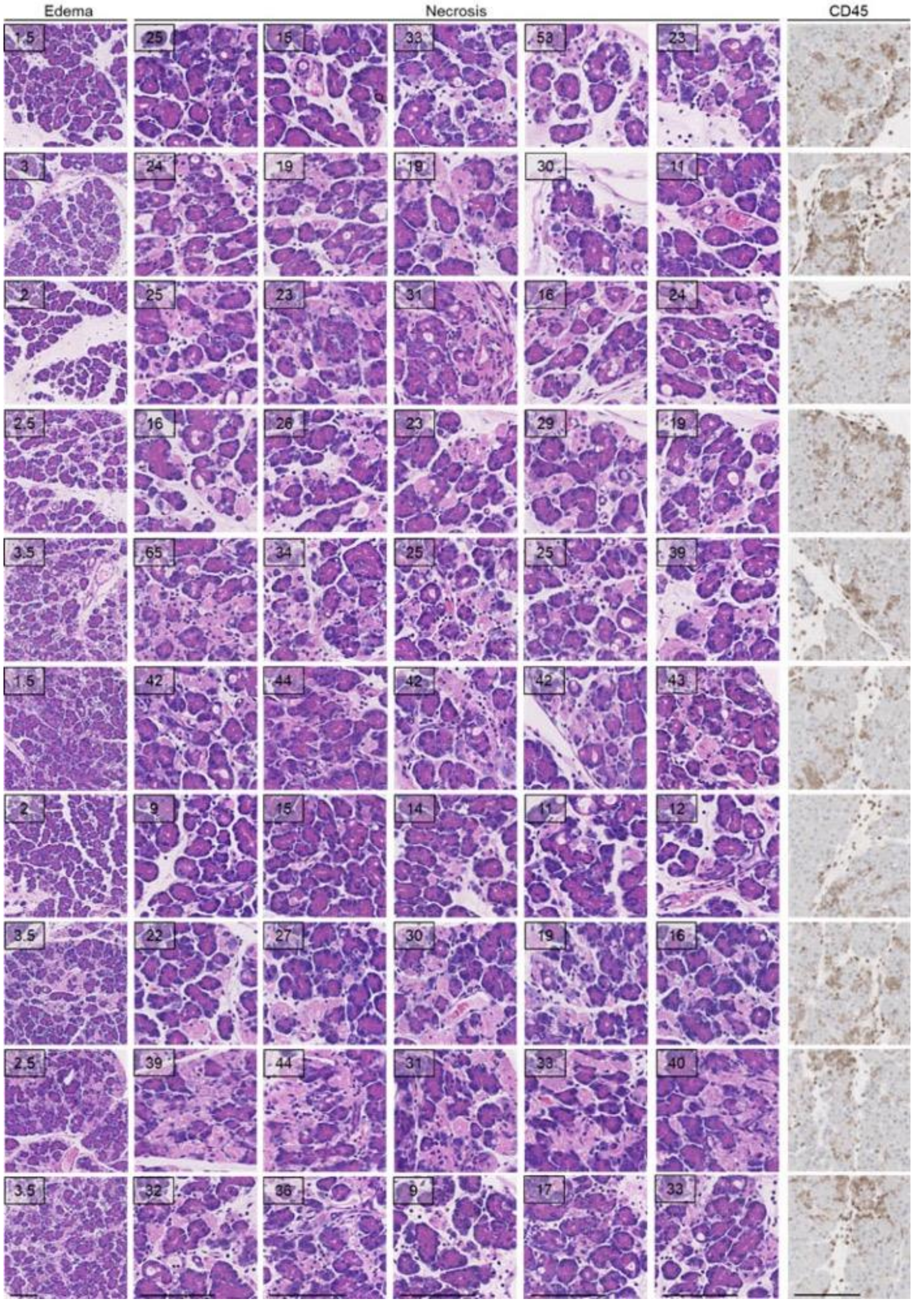

Supplementary Fig. 9. Histopathological analysis of pancreatic sections from MΦ-NP-treated CAE-AP mice. Edema score and counts of necrotic acinar cells were labeled on each image. Each row of images included selected areas from the same pancreas. Scale bars = 100 μm. In the study, n = 10 mice for MΦ-NP treatment group. The study was repeated with twice independently with similar results.

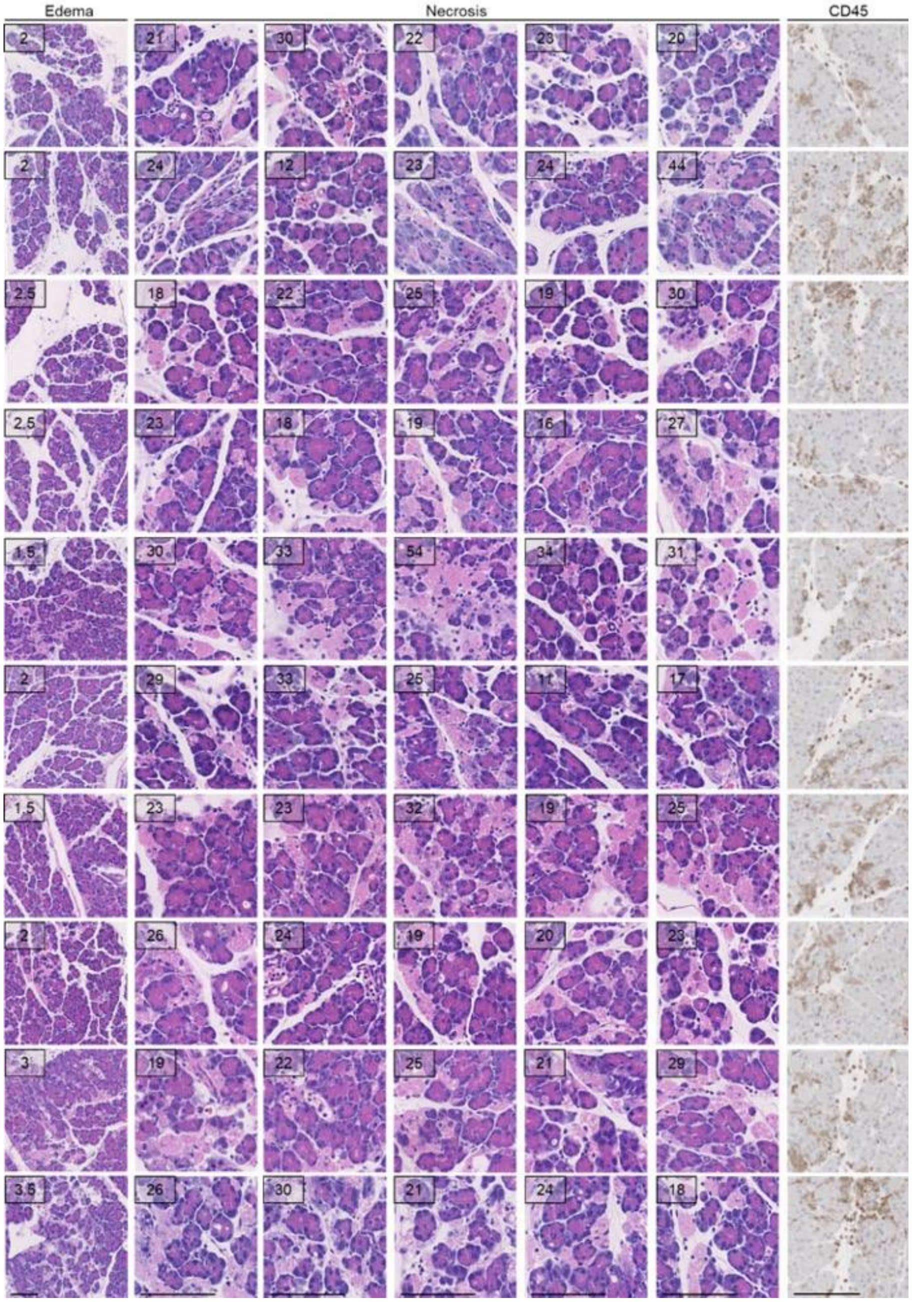

Supplementary Fig. 10. Histopathological analysis of pancreatic sections from MΦ-NP(mel)-treated CAE-AP mice. Edema score and counts of necrotic acinar cells were labeled on each image. Each row of images included selected areas from the same pancreas. Scale bars = 100 μm. In the study, n = 10 mice for MΦ-NP(mel) treatment group. The study was repeated twice independently with similar results.

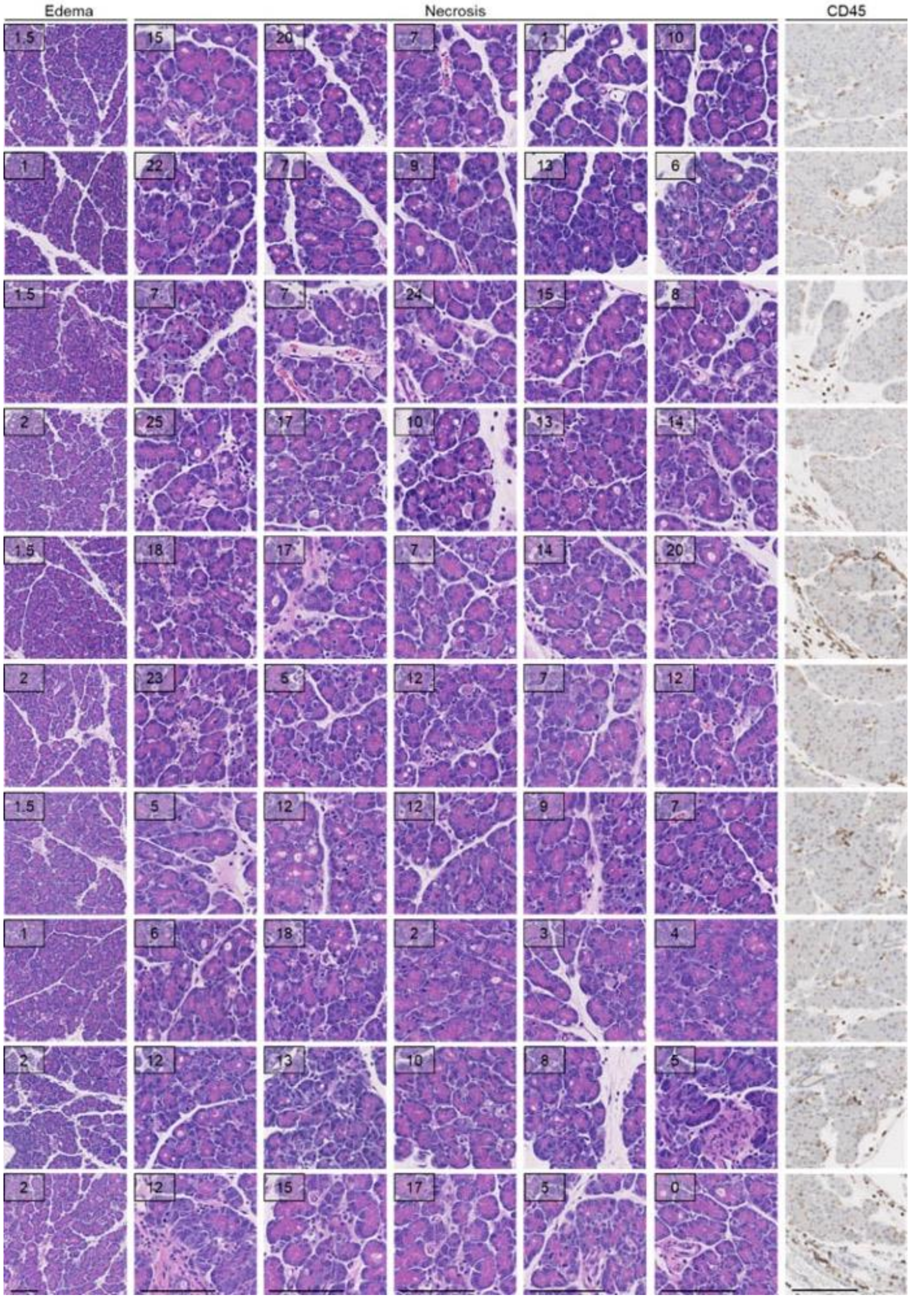

Supplementary Fig. 11. Histopathological analysis of pancreatic sections from MΦ-NP(MJ)-treated CAE-AP mice. Edema score and counts of necrotic acinar cells were labeled on each image. Each row of images included selected areas from the same pancreas. Scale bars = 100 μm. In the study, n = 10 mice for MΦ-NP(MJ) treatment group. The study was repeated twice independently with similar results.

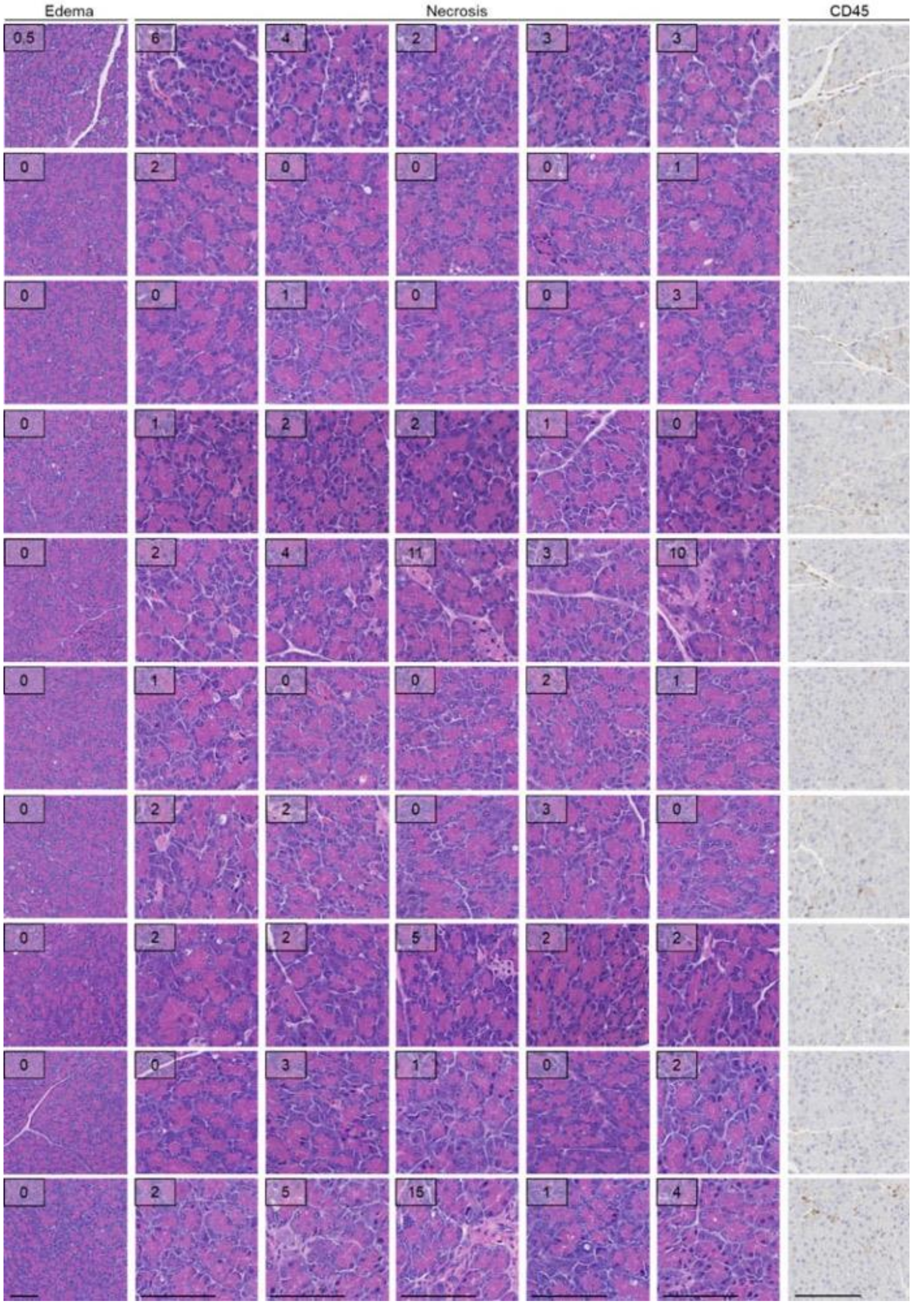

Supplementary Fig. 12. Histopathological analysis of pancreatic sections from MΦ-NP(L&K)-treated CAE-AP mice. Edema score and counts of necrotic acinar cells were labeled on each image. Each row of images included selected areas from the same pancreas. Scale bars = 100 μm. In the study, n = 10 mice for MΦ-NP(L&K) groups. The study was repeated twice independently with similar results.

Pancreatic tissues from CAE-AP mice were fixed, sectioned, stained with anti-CD45 antibody, and probed with biotinylated anti-rabbit IgG antibody for chromagen development. Stained sections were visualized by a Hamamatsu NanoZoomer 2.0-HT slide scanning system.

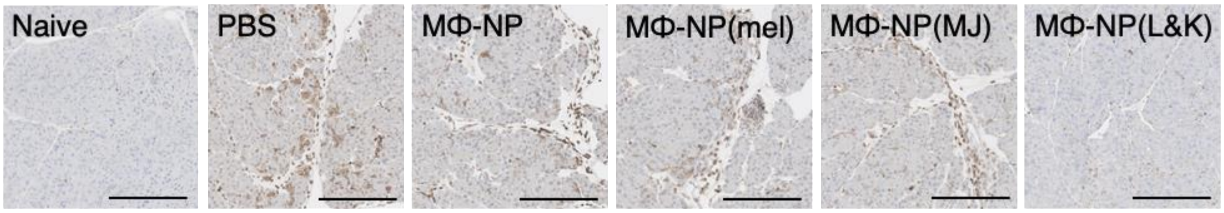

Supplementary Fig. 13. Representative images of anti-CD45 staining on pancreas sections from CAE-AP mice collected 24 h after receiving the treatments. In all groups, n = 10 mice. Scale bars, 200 μm.

To study the effect of CDE-diet intake period on mouse survival rate, 4-week-old CD-1 female mice were fasted on day 0 and randomly divided into 4 groups. Mice in group 1 were fed with normal laboratory diet from day 1 to study endpoint. Mice in groups 2, 3, and 4 were fed with CDE-diet on day 1, days 1 and 2, or days 1 to 3, respectively, followed by normal laboratory diet until study endpoint. Survival was monitored for 10 days.

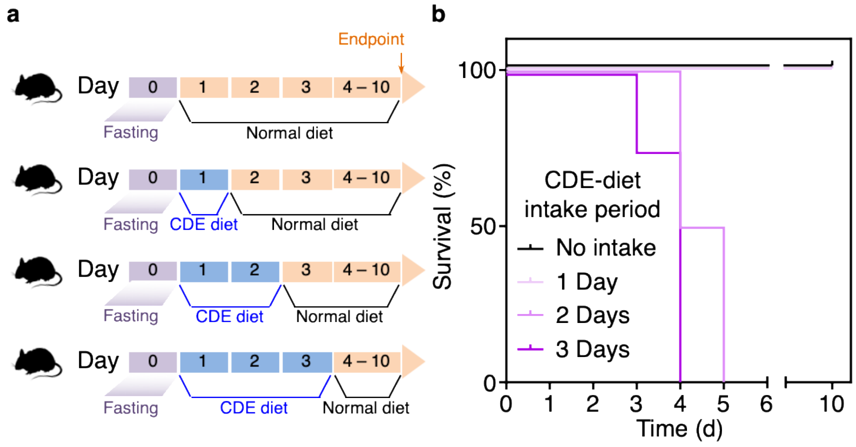

Supplementary Fig. 14. Effect of choline-deficient diet with DL-ethionine (CDE-diet) feeding period on animal survival rate in a mouse model of CDE-diet-induced acute pancreatitis (CDE-AP). **a**, The study protocol of evaluating the effect of CDE-diet intake period on animal survival rate. **b**, Animal survival rate was found to be correlated with CDE-diet intake period. A diet intake duration of 3 days was chosen for the therapeutic studies to minimize the variations in survival rates. In all groups, n = 4 mice. Source data are provided as a Source Data file for Supplementary Fig. 14b.

To study the effect of escalating doses of MΦ-NP(L&K) against CDE-AP, 200 µl of MΦ-NP(L&K) (40 or 80 mg kg^{-1}) was administered intravenously through the tail vein on days 1, 2, and 3 of the studies. Sterile PBS (200 µl) was administered intravenously to mice at the same time as controls. Whole blood of CDE-AP mice was collected on day 3 (~100 µl whole blood per mouse) of the study. CDE-AP mouse serum samples were derived and stored following the procedures described above.

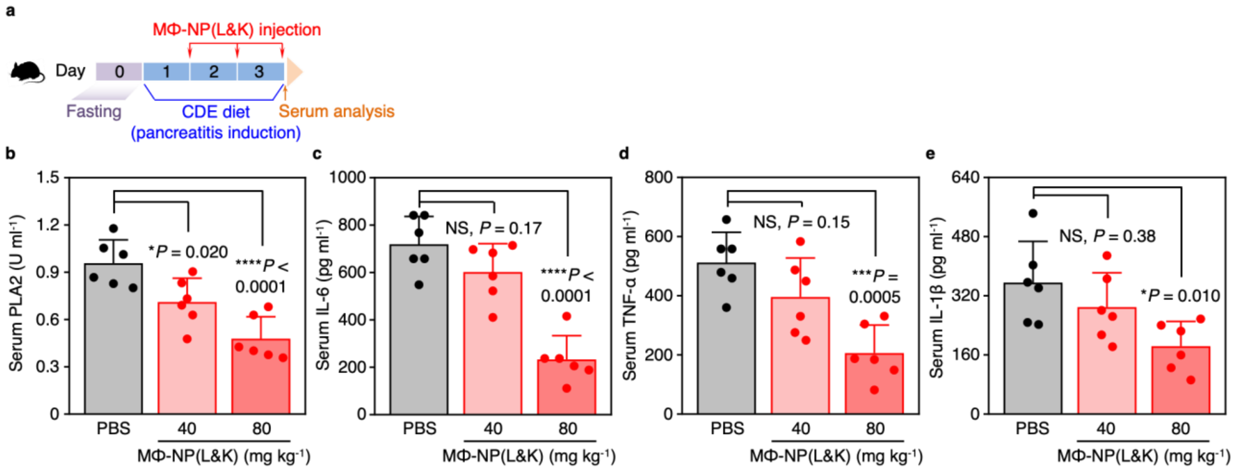

Supplementary Fig. 15. Effect of MΦ-NP(L&K) dosage on alleviating PLA2-induced inflammatory response in a mouse model of CDE-AP. **a**, The study protocol of pancreatitis induction and treatment with MΦ-NP(L&K) (40 or 80 mg kg^{-1}). **b**, PLA2 activity at day 3 after initial CDE-diet intake in serum of CDE-AP mice treated with different doses of MΦ-NP(L&K). **c**-**e**, Concentrations of key inflammatory cytokines including IL-6 (**c**), TNF-α (**d**), and IL-1β (**e**) at 4 h after initial CDE-diet intake in the serum of CDE-AP mice treated with different doses of MΦ-NP(L&K). For all datasets, statistical analysis was performed using one-way ANOVA with Dunnett's post hoc analysis. NS, not significant. Data presented as mean ± s.d. In all datasets, n = 6 mice treated with the same batch of MΦ-NP(L&K). Source data are provided as a Source Data file for Supplementary Fig. 15**b**-**e**.

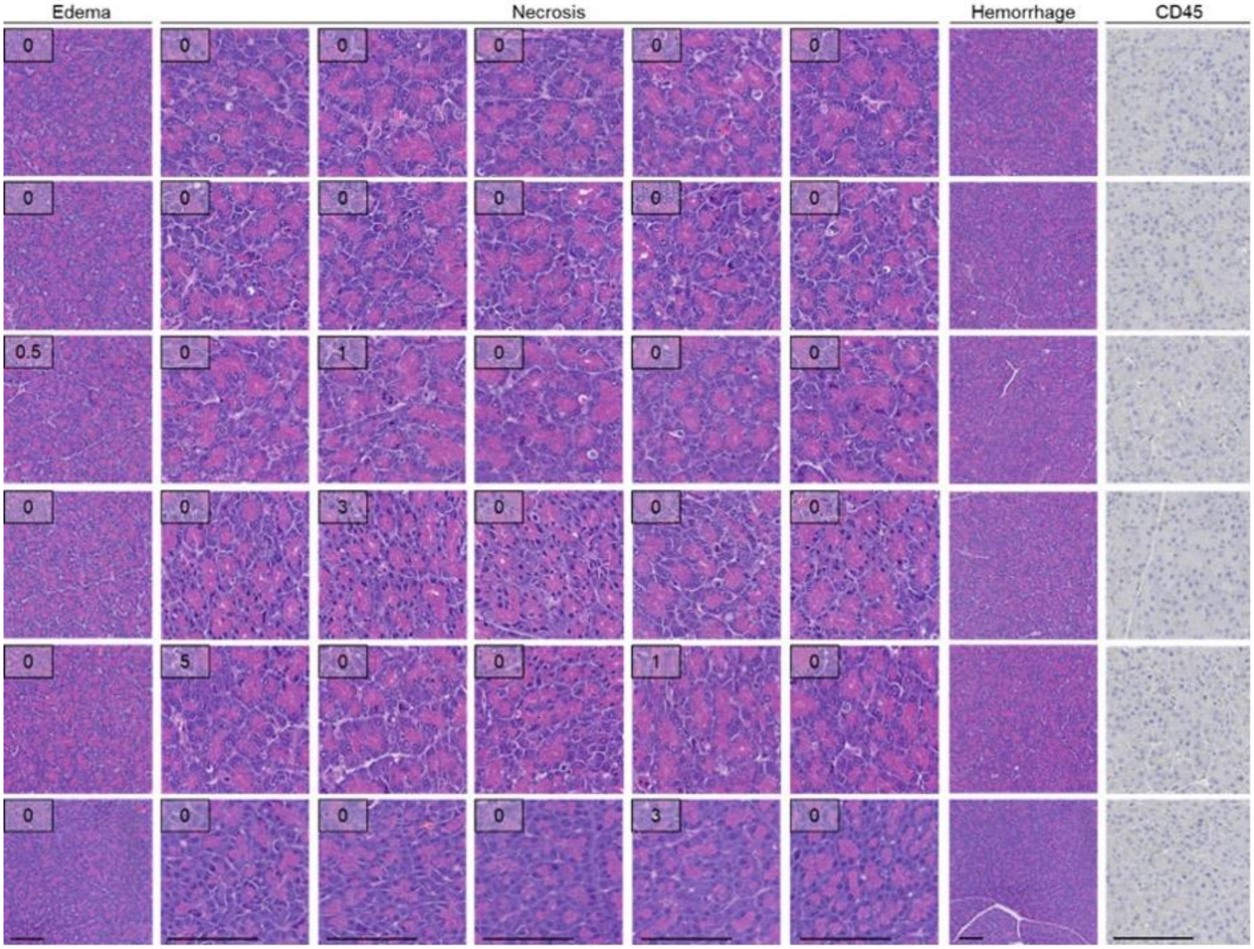

Supplementary Fig. 16. Histopathological analysis of pancreatic sections from healthy naïve mice in the CDE-AP study. Edema score and counts of necrotic acinar cells were labeled on each image. Hemorrhagic areas were undetected in all pancreatic sections. Each row of images included selected areas from the same pancreas. Scale bars = 100 μm. In the study, n = 6 mice for healthy control group. The study was repeated twice independently with similar results.

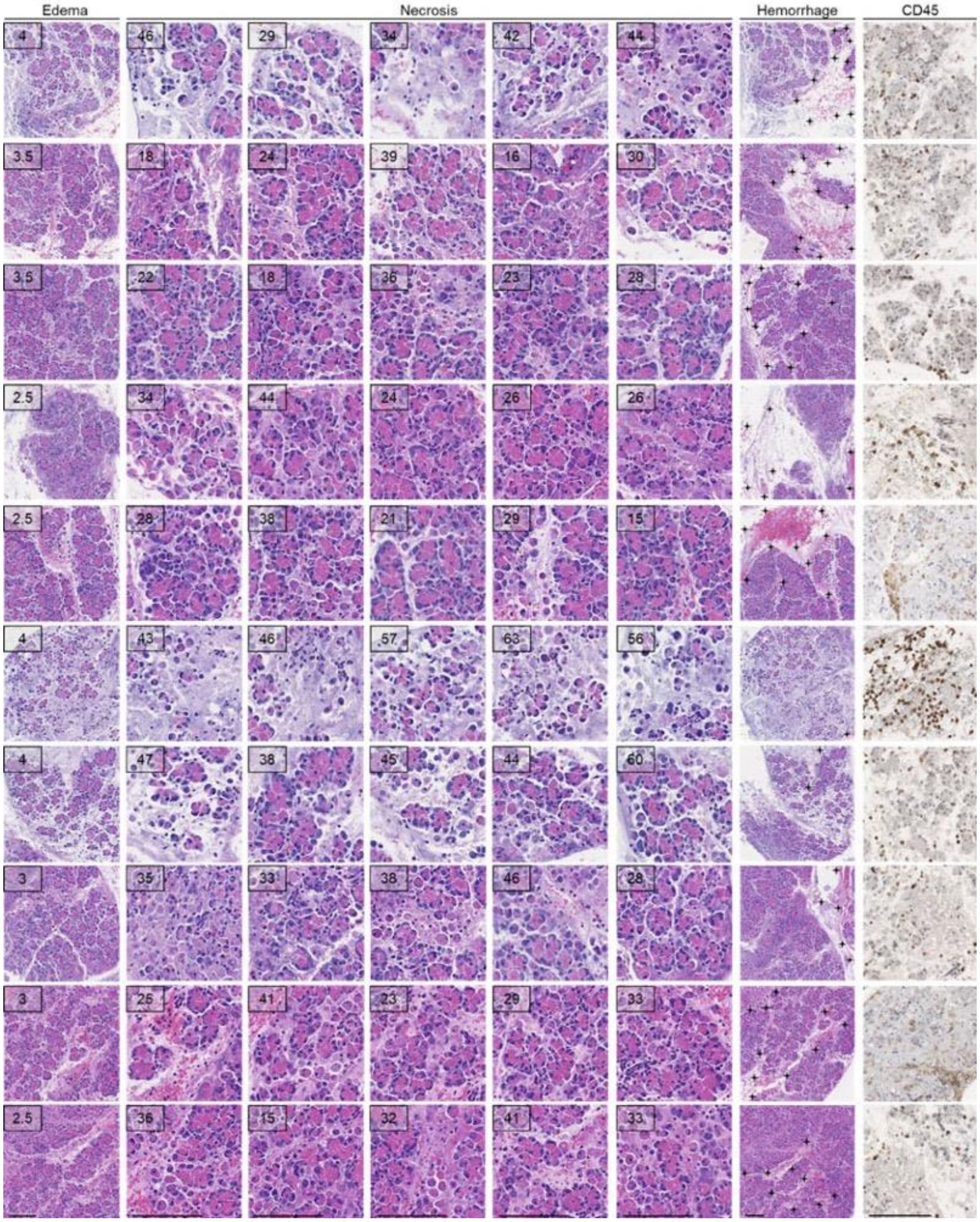

Supplementary Fig. 17. Histopathological analysis of pancreatic sections from PBS-treated CDE-AP mice. Edema score and counts of necrotic acinar cells were labeled on each image. Hemorrhagic areas were indicated with stars. Each row of images included selected areas from the same pancreas. Scale bars = 100 μm. In the study, n = 10 mice for PBS treatment group. The study was repeated twice independently with similar results.

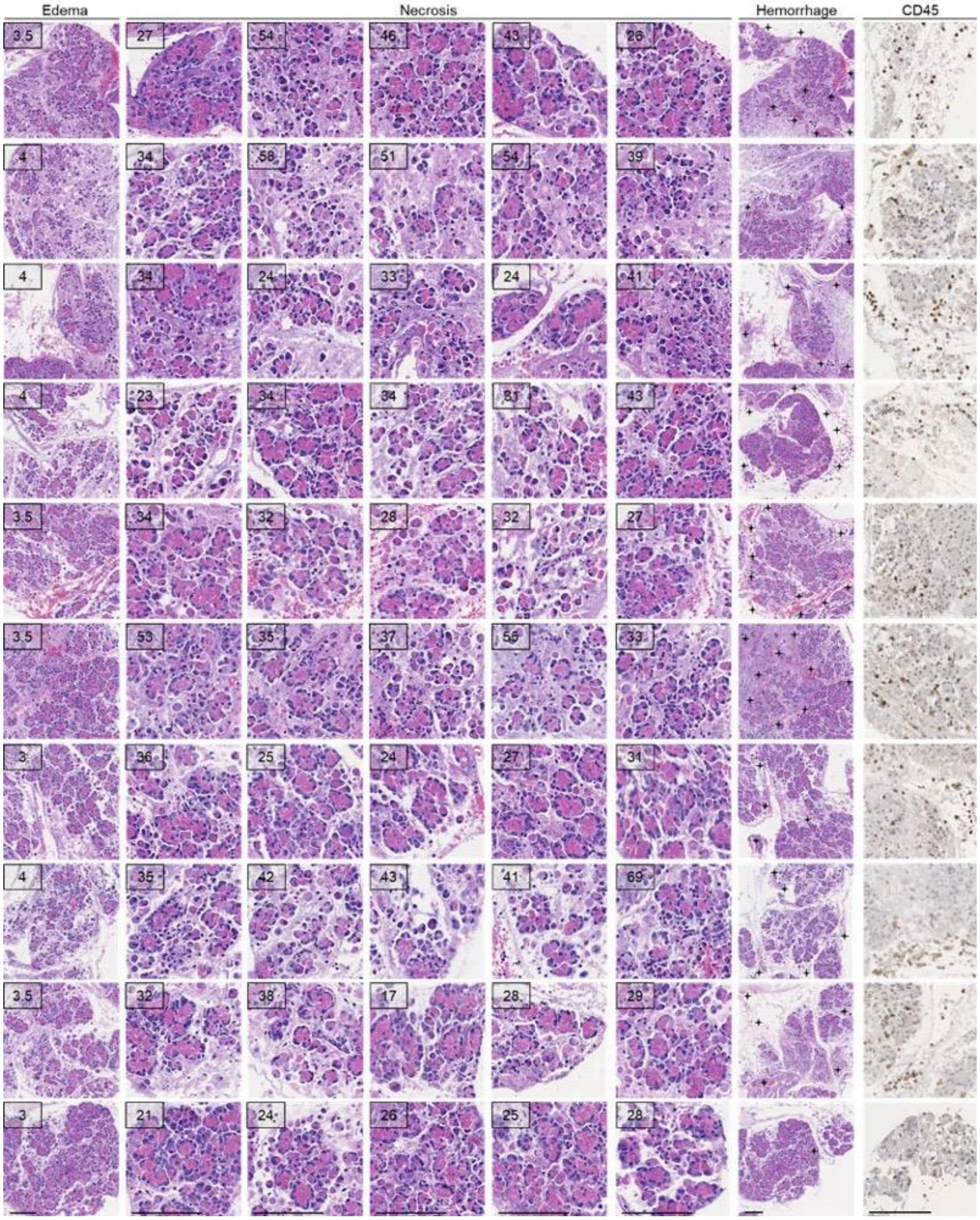

Supplementary Fig. 18. Histopathological analysis of pancreatic sections from MΦ-NP-treated CDE-AP mice. Edema score and counts of necrotic acinar cells were labeled on each image. Hemorrhagic areas were indicated with stars. Each row of images included selected areas from the same pancreas. Scale bars = 100 μm. In the study, n = 10 mice for MΦ-NP treatment group. The study was repeated twice independently with similar results.

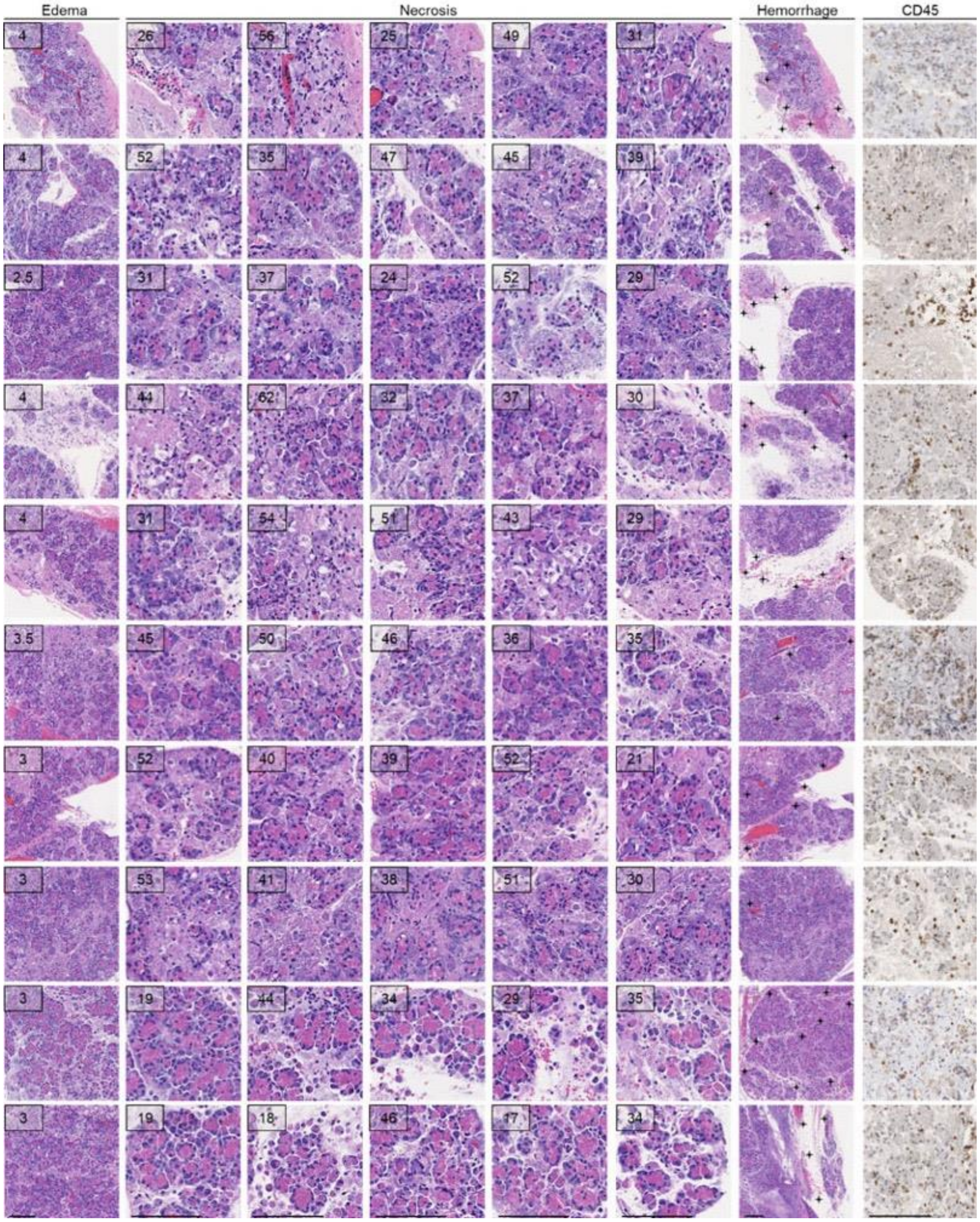

Supplementary Fig. 19. Histopathological analysis of pancreatic sections from MΦ-NP(mel)-treated CDE-AP mice. Edema score and counts of necrotic acinar cells were labeled on each image. Hemorrhagic areas were indicated with stars. Each row of images included selected areas from the same pancreas. Scale bars = 100 μm. In the study, n = 10 mice for MΦ-NP(mel) treatment group. The study was repeated twice independently with similar results.

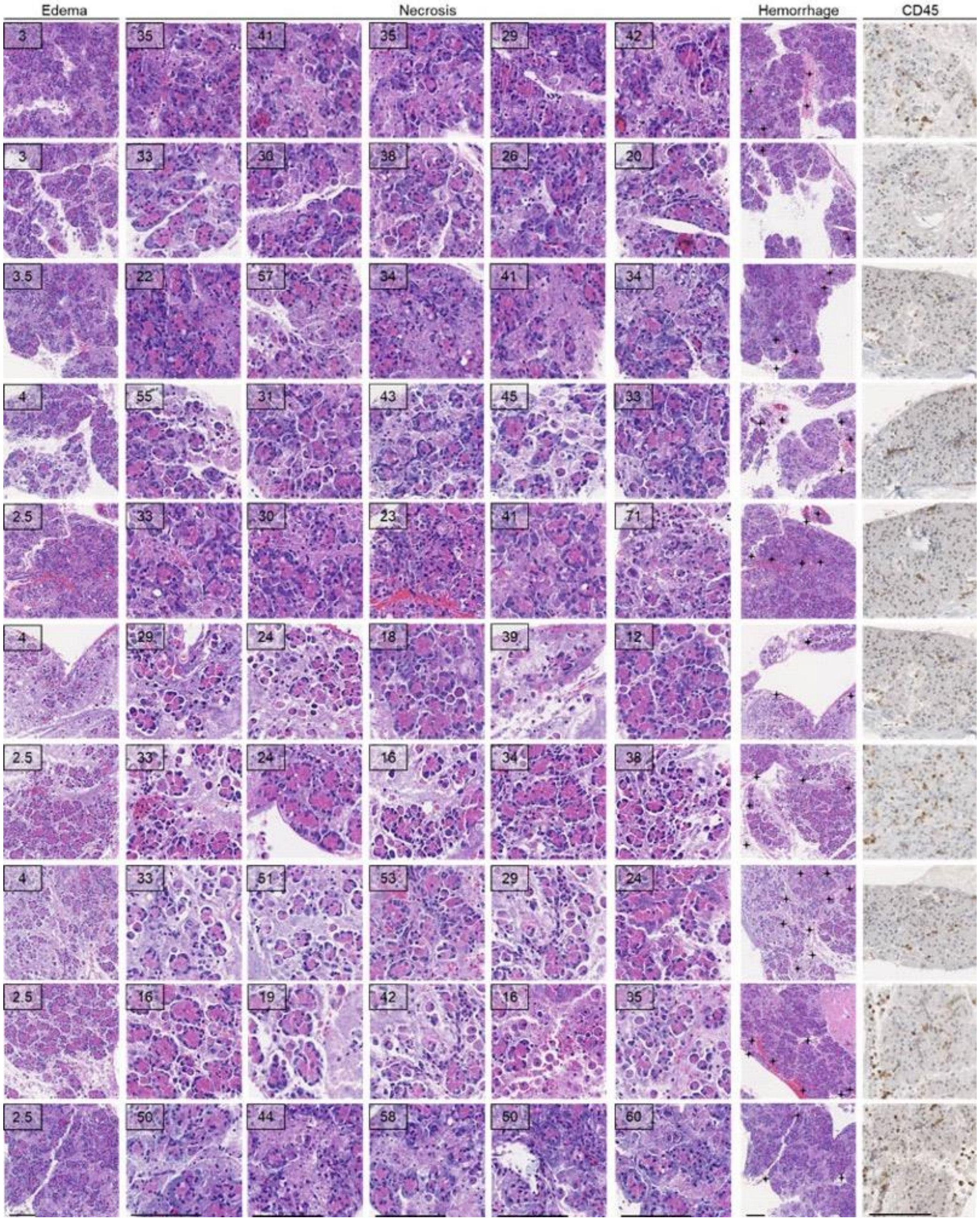

Supplementary Fig. 20. Histopathological analysis of pancreatic sections from MΦ-NP(MJ)-treated CDE-AP mice. Edema score and counts of necrotic acinar cells were labeled on each image. Hemorrhagic areas were indicated with stars. Each row of images included selected areas from the same pancreas. Scale bars = 100 μm. In the study, n = 10 mice for MΦ-NP(MJ) treatment group. The study was repeated twice independently with similar results.

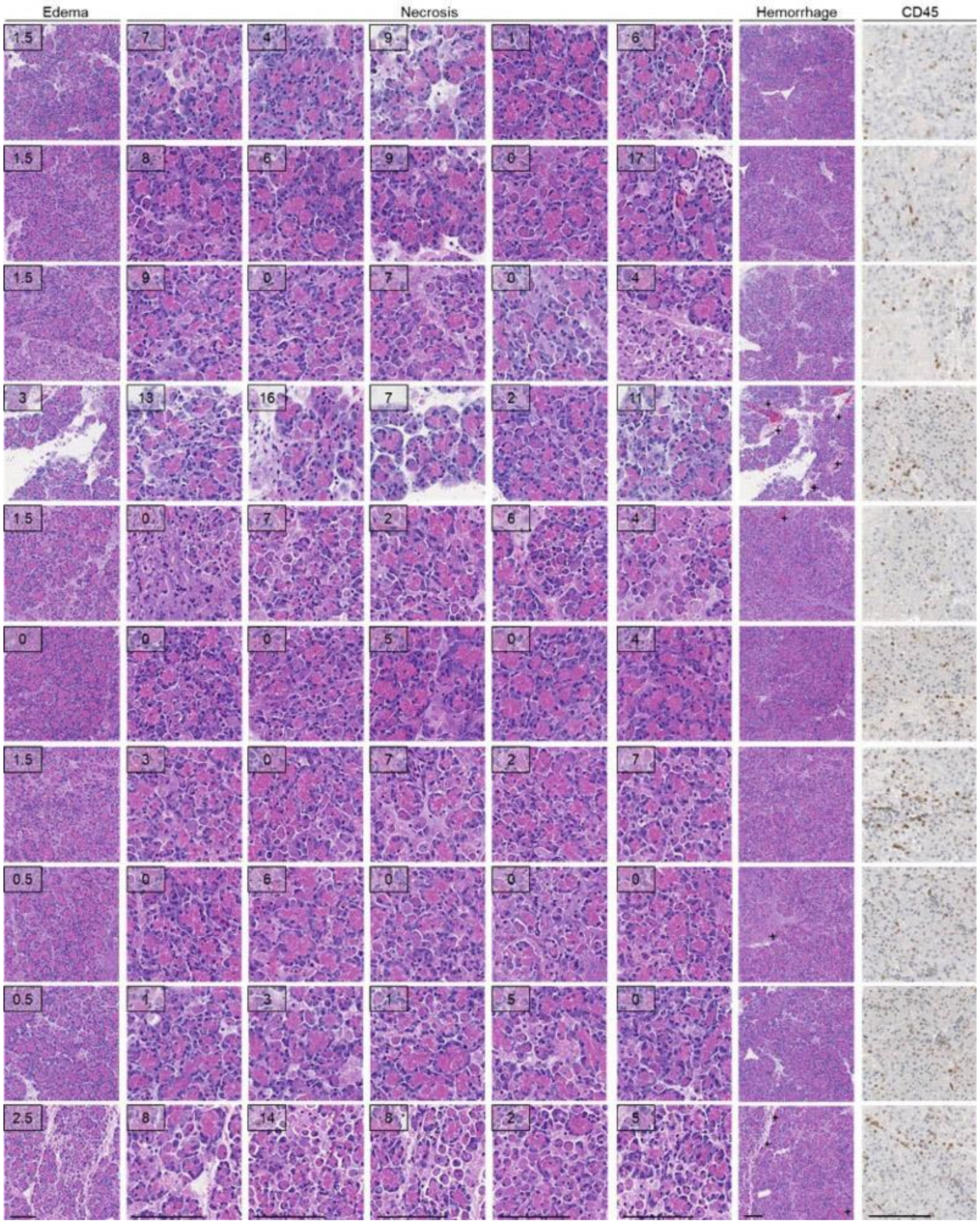

Supplementary Fig. 21. Histopathological analysis of pancreatic sections from MΦ-NP(L&K)-treated CDE-AP mice. Edema score and counts of necrotic acinar cells were labeled on each image. Hemorrhagic areas were indicated with stars and were undetected in some sections. Each row of images included selected areas from the same pancreas. Scale bars = 100 µm. In the study, n = 10 mice for MΦ-NP(L&K) treatment group. The study was repeated twice independently with similar results.

Pancreatic tissues from CDE-AP mice were fixed, sectioned, stained with anti-CD45 antibody, and probed with biotinylated anti-rabbit IgG antibody for chromagen development. Stained sections were visualized by a Hamamatsu NanoZoomer 2.0-HT slide scanning system.

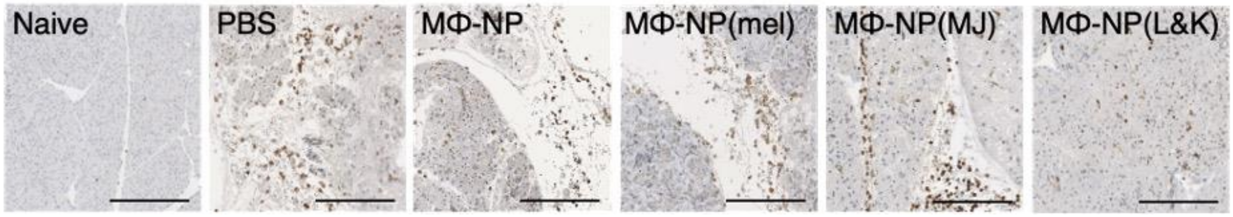

Supplementary Fig. 22. Representative images of anti-CD45 staining on pancreas sections from CDE-AP mice collected 24 h after receiving the treatments. In all groups, n = 10 mice. Scale bars, 200 μm.

Supplementary Table 1.

Characteristics and quality controls of source cells and MΦ-NP(L&K) batches

**Source cells**	**Target range**
**J774A.1**	
Membrane yield	100-125 million cells / mg protein
Mycoplasma testing (every 2 months)	Negative
**THP-1**	
Membrane yield	150-200 million cells / mg protein
Mycoplasma testing (every 2 months)	Negative

**MΦ-NP(L&K)**	**Target range**
Hydrodynamic size in 1 × PBS (nm)	100-130
Polydispersity index (PDI) in 1 × PBS	< 0.2
MJ-33 loading yield (% membrane protein weight)	9.4 ± 0.4
Melittin loading verification (hemolytic activity of MΦ-NP(L&K) supernatant)	< 2% hemolysis

Blood chemistry marker	P value [MΦ-NP(L&K) vs. PBS]	Significance
Albumin (ALB)	0.65	NS
Alkaline phosphatase (ALP)	0.33	NS
Alanine transaminase (ALT)	0.50	NS
Amylase (AMY)	0.47	NS
Total bilirubin (TBIL)	0.42	NS
Blood urea nitrogen (BUN)	0.27	NS
Calcium (CA)	0.47	NS
Phosphorus (PHOS)	0.72	NS
Creatine (CRE)	0.74	NS
Glucose (GLU)	0.28	NS
Sodium (NA+)	0.23	NS
Potassium (K+)	0.92	NS
Total protein (TP)	0.86	NS
Globulin (calculated) (GLOB)	1.00	NS

Supplementary Table 2. Statistical comparison of blood chemistry markers in mice treated with MΦ-NP(L&K) or PBS. The statistical analysis was performed by using the two-tailed paired Student's t-test with Microsoft Excel, followed by Bonferroni correction for multiple comparisons. Significance is assigned when $P < \alpha/n = 0.05/14 = 0.0036$. NS, not significant.
