# Supplementary material for: Lure-and-kill macrophage nanoparticles alleviate the severity of experimental acute pancreatitis
Source: Nat Commun. 2021 Jul 6;12:4136. doi: 10.1038/s41467-021-24447-4 (PMC8260623; doi:10.1038/s41467-021-24447-4)
Supplement: Supplementary file 2 — Reporting Summary [file 41467_2021_24447_MOESM2_ESM.pdf]

nature research

Corresponding author(s): Liangfang Zhang, NCOMMS-21-02038A

Last updated by author(s): May 21st, 2021

Reporting Summary

Nature Research wishes to improve the reproducibility of the work that we publish. This form provides structure for consistency and transparency in reporting. For further information on Nature Research policies, see our Editorial Policies and the Editorial Policy Checklist.

Statistics

For all statistical analyses, confirm that the following items are present in the figure legend, table legend, main text, or Methods section.

n/a	Confirmed	
☐	☒	The exact sample size (*n*) for each experimental group/condition, given as a discrete number and unit of measurement
☐	☒	A statement on whether measurements were taken from distinct samples or whether the same sample was measured repeatedly
☐	☒	The statistical test(s) used AND whether they are one- or two-sided<br>*Only common tests should be described solely by name; describe more complex techniques in the Methods section.*
☐	☒	A description of all covariates tested
☒	☐	A description of any assumptions or corrections, such as tests of normality and adjustment for multiple comparisons
☐	☒	A full description of the statistical parameters including central tendency (e.g. means) or other basic estimates (e.g. regression coefficient) AND variation (e.g. standard deviation) or associated estimates of uncertainty (e.g. confidence intervals)
☐	☒	For null hypothesis testing, the test statistic (e.g. *F*, *t*, *r*) with confidence intervals, effect sizes, degrees of freedom and *P* value noted<br>*Give P values as exact values whenever suitable.*
☒	☐	For Bayesian analysis, information on the choice of priors and Markov chain Monte Carlo settings
☒	☐	For hierarchical and complex designs, identification of the appropriate level for tests and full reporting of outcomes
☒	☐	Estimates of effect sizes (e.g. Cohen's *d*, Pearson's *r*), indicating how they were calculated

Our web collection on statistics for biologists contains articles on many of the points above.

Software and code

Policy information about availability of computer code

Data collection	No stand-alone or custom software was used for data collection.
Data analysis	Softwares used in analysis include GraphPad Prism 8, ImageJ (1.52q), FlowJo VX, and Microsoft Excel (Microsoft 365).

For manuscripts utilizing custom algorithms or software that are central to the research but not yet described in published literature, software must be made available to editors and reviewers. We strongly encourage code deposition in a community repository (e.g. GitHub). See the Nature Research guidelines for submitting code & software for further information.

Data

Policy information about availability of data

All manuscripts must include a data availability statement. This statement should provide the following information, where applicable:

- Accession codes, unique identifiers, or web links for publicly available datasets
- A list of figures that have associated raw data
- A description of any restrictions on data availability

The authors declare that all data supporting the findings of this study are available within the paper and its supplementary information files or available from the corresponding author upon reasonable request. Source data are provided with this paper.

Field-specific reporting

Life sciences study design

All studies must disclose on these points even when the disclosure is negative.

Sample size	Standard number of replication is 3 in all in vitro studies. A minimum of 3 independent repeats is required to evaluate significance. Sample size was chosen based on comparable studies in the field and our own previous published studies (Zhang et al., Nano Letter 2020, PMID 32551679; Zhuang et al., Science Advances 2020, doi: 10.1126/sciadv.aaz6108; Zhou et al., Molecular Pharmaceutics 2019, PMID 30753778; Zhang et al., Nature Nanotechnology 2018, PMID 30177807). These numbers give sufficient statistical power to avoid both Type 1 and Type 2 error. For pharmacokinetic and biodistribution studies, we observed minimal variability between mice, and chose n = 6 based on comparable studies (Thamphiwatana et al., PNAS 2017, PMID 29073076; Hu et al., Nature 2015, PMID 26374997; Hu et al., PNAS 2011, PMID 21690347). Systemic toxicity study was performed with n = 3 following previously reported results (Zhuang et al., Nano Letters 2020, PMID 32352801; Zhang et al., Nano Letter 2020, PMID 32551679). For analysis of microscopy images, we observed low variability among naive cells or mice of the same batch and genetic background, and chose 50<n<~200 cells per visual field / organ region, 1<n<5 organ regions per mouse depending on the parameter of interest and the experiment. Studies on mouse models of acute pancreatitis were conducted with n = 10 to give sufficient statistical power and minimize the effect of variability. The sample size is within the range (8<n<12) reported in similar animal models (Fink & Norman, Cytokine 1997, PMID 9417814; Nakamichi et al., Journal of Clinical Investigation 2005, PMID 16239966).
Data exclusions	No data was excluded.
Replication	Experiments were repeated at least twice with independent batches of samples, and all experimental findings were reproducible. Representative results were presented for each experiment.
Randomization	Samples were randomly allocated to corresponding experimental groups. Organisms were cultured and maintained in the same environment and randomly allocated to each group.
Blinding	Histological samples were scored by a blinded subject who was unaware of the type of treatment administered to the animals. Otherwise the investigators were not blinded during experiments and analyses, because all samples were measured and analyzed equivalently within defined experimental groups.

Reporting for specific materials, systems and methods

We require information from authors about some types of materials, experimental systems and methods used in many studies. Here, indicate whether each material, system or method listed is relevant to your study. If you are not sure if a list item applies to your research, read the appropriate section before selecting a response.

Materials & experimental systems

n/a	Involved in the study	
[ ]	[x]	Antibodies
[ ]	[x]	Eukaryotic cell lines
[x]	[ ]	Palaeontology and archaeology
[ ]	[x]	Animals and other organisms
[x]	[ ]	Human research participants
[x]	[ ]	Clinical data
[x]	[ ]	Dual use research of concern

Methods

n/a	Involved in the study	
[x]	[ ]	ChIP-seq
[ ]	[x]	Flow cytometry
[x]	[ ]	MRI-based neuroimaging

Antibodies

Antibodies used	Mouse anti-mouse NF-κB p65 (Cat#sc-166748; Clone#A-8) was purchased from Santa Cruz Biotechnology. FITC anti-mouse IgG (Cat#406001; Clone#405305) was purchased from Biolegend. Rabbit anti-mouse CD45 (Cat#ab10558) and biotinylated goat anti-rabbit IgG H&L (Cat#: ab67720) was purchased from Abcam.
Validation	Each antibody has been verified by the supplier and each lot has been quality tested. Relevant information is available at: mouse anti-mouse NF-κB p65 (https://datasheets.scbt.com/sc-166748.pdf); FITC anti-mouse IgG (https://www.biolegend.com/en-us/products/fitc-anti-mouse-igg-1394); Rabbit anti-mouse CD45 (https://www.abcam.com/cd45-antibody-ab10558.html); Goat anti-rabbit IgG H&L (https://www.abcam.com/arl11-antibody-ab67720.html).

Eukaryotic cell lines

Policy information about cell lines

Cell line source(s)	Murine macrophages (ATCC J774A.1, TIB-67) were purchased from ATCC and cultured in Dulbecco's Modified Eagle's Medium (Corning 10-017-CV) supplemented with Fetal Bovine Serum (Hyclone SH30541.03) and Pen-Strep (Gibco 15140122). Human monocytes (ATCC THP-1, TIB-202) were purchased from ATCC and cultured in RPMI 1640 Medium (Gibco 11875093) supplemented with Fetal Bovine Serum (Hyclone SH30541.03) and Pen-Strep (Gibco 15140122).
Authentication	Cells were used without modification once received from the supplier and therefore were not authenticated.

Mycoplasma contamination	All cell lines were tested negative for Mycoplasma contamination every month.
Commonly misidentified lines (See ICLAC register)	No commonly misidentified cell lines were used in the study.

Animals and other organisms

Policy information about studies involving animals; ARRIVE guidelines recommended for reporting animal research

Laboratory animals	CD-1 female mice (6-week-old or 4-week-old) were used in the study.
Wild animals	The study did not involve wild animals.
Field-collected samples	The study did not involve samples collected from the field.
Ethics oversight	All animal experiments were performed in accordance with NIH guidelines and approved by the Institutional Animal Care and Use Committee (IACUC) of UCSD.

Note that full information on the approval of the study protocol must also be provided in the manuscript.

Flow Cytometry

Plots

Confirm that:

- [x] The axis labels state the marker and fluorochrome used (e.g. CD4-FITC).
- [x] The axis scales are clearly visible. Include numbers along axes only for bottom left plot of group (a 'group' is an analysis of identical markers).
- [x] All plots are contour plots with outliers or pseudocolor plots.
- [x] A numerical value for number of cells or percentage (with statistics) is provided.

Methodology

Sample preparation	Pancreatic acini were isolated from healthy ICR mice (Harlan Laboratory, female, 6-weeks-old) by enzymatic digestion of the pancreatic tissue. Pancreatic acini were used without further modification. Details on cell death pathway staining using PI (Biolegend #421301) and FITC Annexin-V (Biolegend #640905) are provided in Methods.
Instrument	Becton Dickinson FACSCanto-II flow cytometer.
Software	FlowJo VX
Cell population abundance	Pancreatic acinar cells were isolated following previously validated method for isolation of pure population of primary acinar cells from mouse pancreas, without the presence of contaminating cell types (doi: 10.3791/50514). The current study adopted the aforementioned isolation protocol without modifications and population abundance was not tested.
Gating strategy	For in vitro study, preliminary FSC/SSC gates were determined by naive pancreatic acini sample. Staining boundaries were determined by comparing the fluorescence signals of naive pancreatic acini (Annexin-V-negative, PI-negative) with pancreatic acini treated with concentrated Cae-AP mouse serum. (Please see Supplementary Fig. 4A)

- [x] Tick this box to confirm that a figure exemplifying the gating strategy is provided in the Supplementary Information.
